# Nested Formation of Calcium Carbonate Polymorphs in a Bacterial Surface Membrane with a Graded Nanoconfinement: An Evolutionary Strategy to Ensure Bacterial Survival

**DOI:** 10.1021/acsbiomaterials.1c01280

**Published:** 2022-01-07

**Authors:** Paul Simon, Wolfgang Pompe, Denise Gruner, Elena Sturm, Kai Ostermann, Sabine Matys, Manja Vogel, Gerhard Rödel

**Affiliations:** † Max Planck Institute for Chemical Physics of Solids, Nöthnitzer Straße 40, 01187 Dresden, Germany; ‡ Institute of Materials Science, Technische Universität Dresden, Helmholtzstraße 7, 01069 Dresden, Germany; § Institute of Genetics, Technische Universität Dresden, Zellescher Weg 20b, 01217 Dresden, Germany; ∥ Helmholtz Institute Freiberg for Resource Technology, Helmholtz-Zentrum Dresden-Rossendorf, Bautzener Landstraße 400, 01328 Dresden, Germany; ⊥ Polymeric Microsystems, Technische Universität Dresden, Helmholtzstraße 100, 01069 Dresden, Germany; # Physical Chemistry, University of Konstanz, POB 714, D-78457 Konstanz, Germany

**Keywords:** S-layer, peptidoglycan layer, nanostructures, calcium carbonate, forced biomineralization, HR-TEM

## Abstract

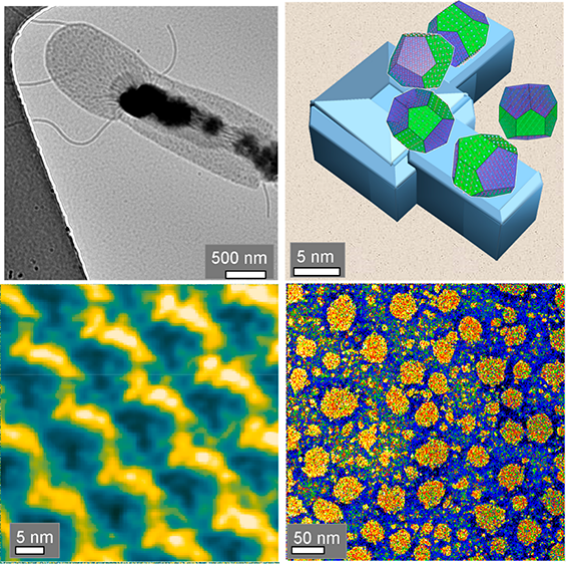

It is the intention of this study to elucidate the nested formation of calcium carbonate polymorphs or polyamorphs in the different nanosized compartments. With these observations, it can be concluded how the bacteria can survive in a harsh environment with high calcium carbonate supersaturation. The mechanisms of calcium carbonate precipitation at the surface membrane and at the underlying cell wall membrane of the thermophilic soil bacterium *Geobacillus stearothermophilus* DSM 13240 have been revealed by high-resolution transmission electron microscopy and atomic force microscopy. In this Gram-positive bacterium, nanopores in the surface layer (S-layer) and in the supporting cell wall polymers are nucleation sites for metastable calcium carbonate polymorphs and polyamorphs. In order to observe the different metastable forms, various reaction times and a low reaction temperature (4 °C) have been chosen. Calcium carbonate polymorphs nucleate in the confinement of nanosized pores (⌀ 3–5 nm) of the S-layer. The hydrous crystalline calcium carbonate (ikaite) is formed initially with [110] as the favored growth direction. It transforms into the anhydrous metastable vaterite by a solid-state transition. In a following reaction step, calcite is precipitated, caused by dissolution of vaterite in the aqueous solution. In the larger pores of the cell wall (⌀ 20–50 nm), hydrated amorphous calcium carbonate is grown, which transforms into metastable monohydrocalcite, aragonite, or calcite. Due to the sequence of reaction steps via various metastable phases, the bacteria gain time for chipping the partially mineralized S-layer, and forming a fresh S-layer (characteristic growth time about 20 min). Thus, the bacteria can survive in solutions with high calcium carbonate supersaturation under the conditions of forced biomineralization.

## I Introduction

The thermophilic soil Gram-positive bacterium *Geobacillus stearothermophilus* DSM 13240 (*G. stearothermophilus*) can be classified into the archaebacteria, as one of the first forms of life on earth. As with other similar bacteria, it can also exist under extreme environmental conditions (higher or deeper temperature, higher salt concentration). Thus, the growth of calcium carbonate polymorphs at the cell membrane of *G. stearothermophilus* provides the option to study processes of forced biomineralization.^1^ The cell wall of Gram-positive bacteria composed of a thicker peptidoglycan layer and often covered with a two-dimensional protein arrangement, the so-called S-layer, can act as an effective place for nucleation and growth of minerals known as biologically induced mineralization.^2−4^ Particularly, positively charged metal ions can be precipitated at such structures.^3,5,6^ Recently, special processes of forced biomineralization have been studied in more detail, when a high concentration of metallic ions leads to the development of diverse biomineralized structures contributing to the survival of extremophiles.^1^ Forced biomineralization is focused on the following phenomena:

- The biomineralization of iron-, silica-, and calcium-based phases at extreme environmental conditions
- The survival strategies of prokaryotes and eukaryotes using the protective advantages of biomineralization due to functionalization of their cell envelopes
- The mechanisms controlling fossilization, as well as exceptional preservation of organic templates, which strongly bind to the mineral surface
- The underlying mechanisms used by diverse extremophiles and polyextremophiles to exhibit extreme cold (cryo), heat (thermo), and pressure (piezo) tolerance^1,7,8^

In the following, it will be shown that the biomineralization of calcium carbonate by *G. stearothermophilus* represents a sound model system for essential features of forced biomineralization. In nature, these thermophile bacteria live not only under normal environmental conditions but also under higher temperatures. For instance, they have been observed in the hot springs of the Yellowstone National Park, in the deep sea,^9^ or in a hydrothermally active volcanic area.^10^ With their tolerance of toxic minerals, for instance, uranium and arsenic compounds, they also exist at mining waste disposal sites.^11^

**Figure 1:**
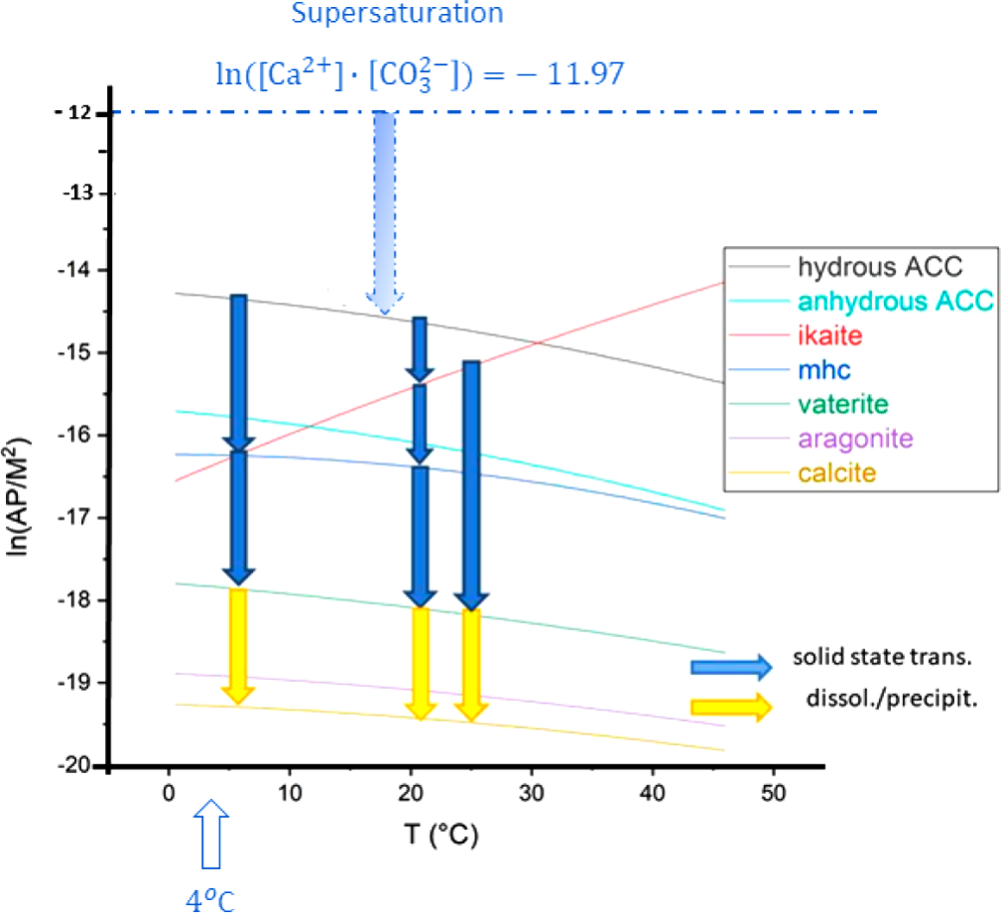
Precipitation diagram of calcium carbonate with possible reaction channels for the formation of metastable calcium carbonate polymorphs under the condition of high supersaturation. The natural logarithm of the activity products of calcium and carbonate ions, ln(AP/*M*^2^), is plotted in dependence on the temperature *T* (°C). The equilibrium curves for the reactions of the various polymorphs have been calculated using equilibrium constants published by Brecevic and Nielsen,^19^ Bischoff et al.,^20^ Plummer and Busenberg,^21^ and Kralj and Brecevic.^22^ The equilibrium curves of amorphous calcium carbonate (ACC·*x*H_2_O) cover a larger range in the diagram depending on the water content *x*.^23,24^

The relevance of bacterial calcium carbonate mineralization in modern and ancient geochemical cycles of C and/or Ca has been extensively studied (e.g., by Görgen et al.,^4^ v. Knorre and Krumbein,^12^ Boquet et al.,^13^ and Uriz et al.^14^). The cell membrane of *G. stearothermophilus* possesses an interesting graded pore size distribution. In the paracrystalline S-layer, nanosized pores (⌀ 3–5 nm) are formed, whereas in the supporting thicker layer of proteoglycans (PG-layer), larger pores (⌀ 20–50 nm) exist. It is the intention of this study to elucidate the nested formation of calcium carbonate polymorphs or polyamorphs in these different nanosized compartments. With these observations, it can be concluded how the bacteria can survive in a harsh environment with high calcium carbonate supersaturation. The *G. stearothermophilus* DSM 13240 (as complete bacteria as well as only the separated self-assembled S-layer) has been mineralized in a highly supersaturated CaCl_2_/Na_2_CO_3_ solution. In the biomineralization of calcium carbonate, in addition to the stable equilibrium phase calcite, often metastable crystalline polymorphs^15^ or polyamorphs^16−18^ are observed. From the precipitation diagram of calcium carbonate, shown in Figure 1, it follows that for the synthesis of the metastable phases a high supersaturation *S_i_* of the calcium and carbonate ions in aqueous solution is needed, as the driving force Δ*G*_*i*_ for the precipitation of a particular polymorph *i* is given by
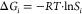
1where *R* is the gas constant and *T* the absolute temperature. *S_i_* depends on the product of the ion activities *a*_Ca^2+^_·*a*__CO__3_^2–^_ and on the thermodynamic solubility *K*_*s*,*i*_ of the polymorph
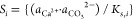
2In the following discussion, we substitute the ion activities by the ion concentrations [Ca^2+^]·[CO_3_^2–^] assuming an ideal solution.

We have selected the well-known replacement reaction of sodium carbonate with calcium chloride to realize such a condition. As derived in Supporting Information (SI), Supplement S1, in a solution of 10 mM CaCl_2_ and 0.1 M Na_2_CO_3_ in distilled water, an activity product of the calcium and carbonate ions AP = [Ca^2+^]·[CO_3_^2–^] with ln(AP/*M*^2^) = −11.97 exists. In order to detect the metastable intermediate products of the replacement reaction with sufficient probability, the reaction temperature of 4 °C has been chosen. At the beginning of the study, there was the open question as to what extent the presence of the biomolecular template could influence the replacement reaction and the formation of the various polymorphs (particularly ikaite). *G. stearothermophilus* is an auspicious candidate for the study of biomineralization of these various metastable phases as it can be assumed that these bacteria can possess full activity under such harsh conditions.

In order to observe the different metastable forms, various reaction times and a low reaction temperature (4 °C) have been chosen.

The various stages of the mineralization process have been investigated by high-resolution transmission electron microscopy (HR-TEM) and atomic force microscopy (AFM). These techniques are complementary to characterize the biomolecular nanostructure (AFM), and the crystalline structure of the precipitated calcium carbonate polymorphs (HR-TEM), quantitatively. Usually, macroscale and mesoscopic methods such as IR, AFM, SEM, or X-ray spectroscopy and X-ray diffraction have been applied to characterize the bacterial cell membrane and its mineralization.^25−34^ To our knowledge, only few publications addressed its biomineralization at the nanoscale.^35−38^

## II Results

**Figure 2:**
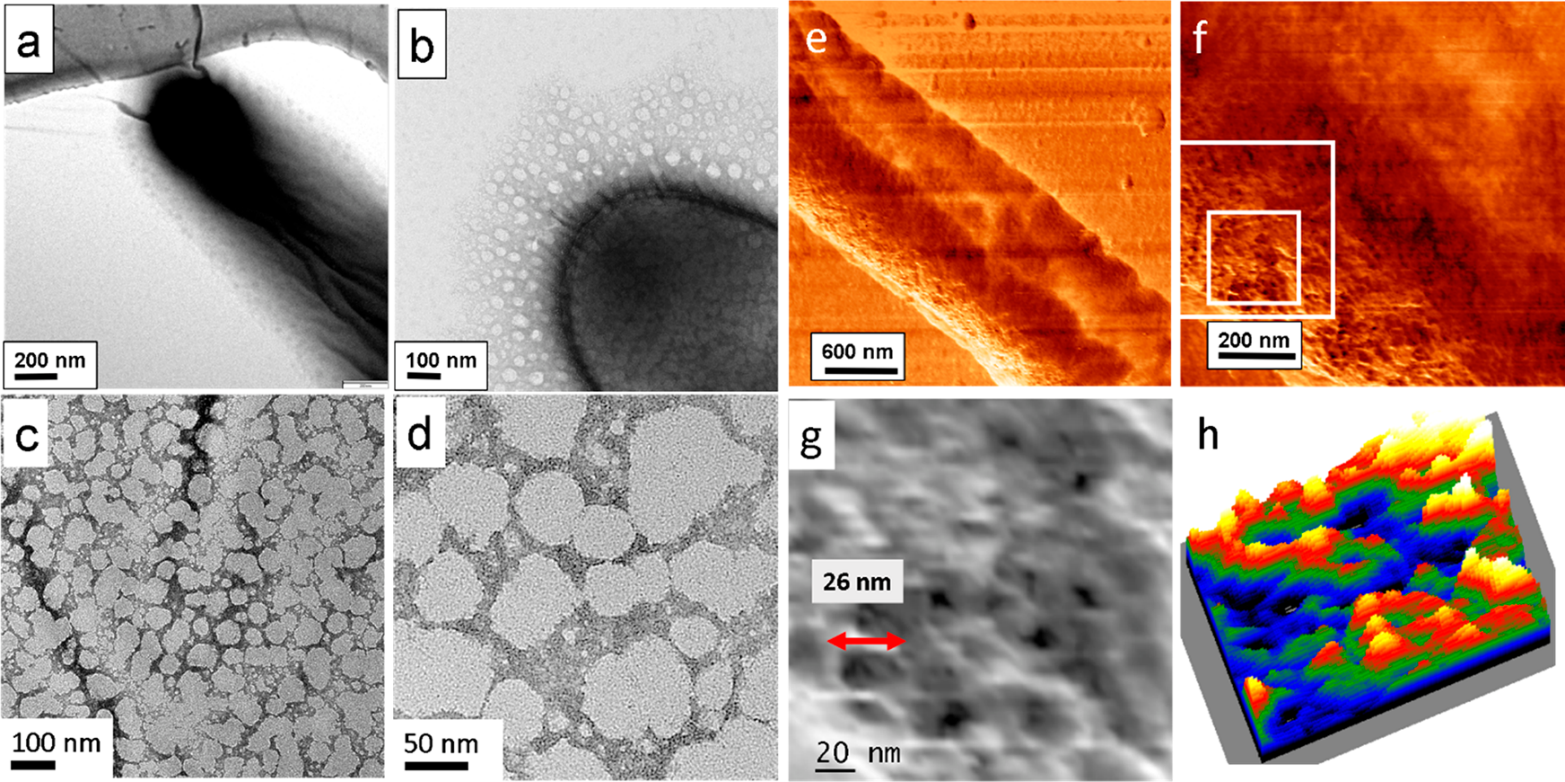
TEM and AFM (in liquid using the AC mode) images of the native bacterium exhibiting a length of 18 μm and a width of 1 μm. (a, b) TEM image of a zoom-in of one end of the native bacterium. (c,d) TEM micrographs of uranyl-stained *G. stearothermophilus* showing large pores. (e) AFM height micrograph of bacterium. (f) AFM phase imaging of a whole bacterium in a cross section in tapping mode depicting regions with different elastic modulus, namely, of the upper S-layer and the underlying peptidoglycan (PG- layer) of the cell wall. (g) Zoomed image of the small white frame in panel f. Larger pores are revealed in the range of 20–50 nm diameter as observed in the TEM images too, see panels b and c. The nanopores shown in panel g are black in this phase image. (h) 3D display of panel g, with larger pores appearing green and blue.

In the present work, the bacterium *G. stearothermophilus* was imaged in the TEM in the dry state. The AFM imaging has been performed in liquid using the AC mode. The length and the width of the bacterium amount to 18 and 1 μm, respectively (Figure 2a,e).

### II.i Nanoporosity of the Bacterial Membrane

In Figure 2, TEM (a–d) and AFM (AC mode) (e–h) images of native and stained bacterium *G. stearothermophilus* are shown. The nonstained cell (Figure 2a,b) displays regions where the bacterial membrane (S-layer linked to the underlying PG-layer of the cell wall) is partially exfoliated from the cell body. The size of the larger pores appears mainly between 20 and 40 nm (Figure 2c,d and SI Figure S2.1) as observed on the uranyl-stained discarded bacterial membrane. Also, smaller pores with a diameter of 3–5 nm occur, and even larger pores are revealed, mostly around 50–100 nm in size, shown in Figure 2d, and these are assumed to be formed by merged pores. AFM phase images of the bacterium (Figure 2e,f) show the topography at a large field of view. Further zoom reveals the presence of large pores in AFM (Figure 2g,h) in accordance with TEM.

**Figure 3:**
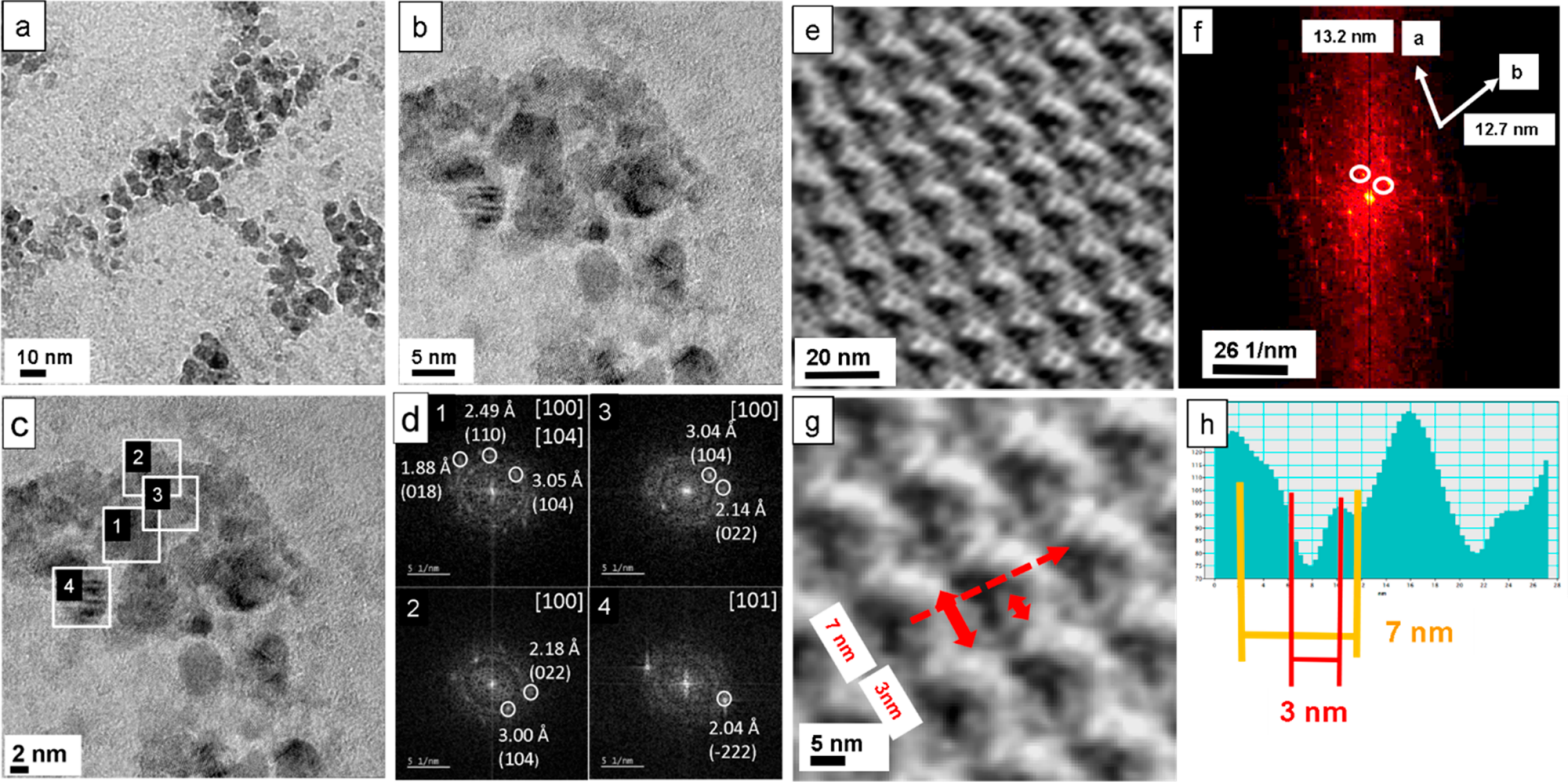
High-resolution structure of a self-assembled layer with the S-layer protein of *G. stearothermophilus*, DSM 13240. (a) Mineralized S-layer patch. (b) Zoomed image of the mineralized aggregates composed of calcite. (c) Regions selected for FFT analysis. (d) Analysis of the high-resolution image in panel c. The FFTs show that only calcite is present after 24 h of incubation, mainly oriented in the [100] direction. (e) AFM overview image. (f) FFT of the AFM image e with *p*2 symmetry. The FFT yields a precise determination of the lattice constants. (g) Zoomed region of AFM image (AC mode). Arrow indicates direction of line profile taken for image h. (h) Evaluation of the height profile showing the dimensions of nanosized pores.

In Figure 3, the highly resolved structure of a self-assembled layer with the S-layer protein of *G. stearothermophilus* is shown. In Figure 3a, a TEM image of the studied S-layer is given, mineralized after 24 h of incubation time. Figure 3b shows a magnified image with individual nanocrystallites. In Figure 3c, regions are marked for FFT analysis presented in Figure 3d. The analysis shows that calcite crystals prevail, mainly oriented in the [100] direction. The overview AFM image of Figure 3e displays the topography of the S-layer. In the fast Fourier transform (FFT) of the AFM image (Figure 3f), the *p*2 symmetry of the S-layer is visible with lattice spacings of 12.7 and 13.2 nm. Figure 3g shows a magnified region of Figure 3e with nanopores of 7 and 3 nm width. The height profile is derived along the red arrow, see Figure 3h. It allows the detailed determination of the pore geometry.

### II.ii Mineralization of a Partially Discarded Bacterial Membrane

**Figure 4:**
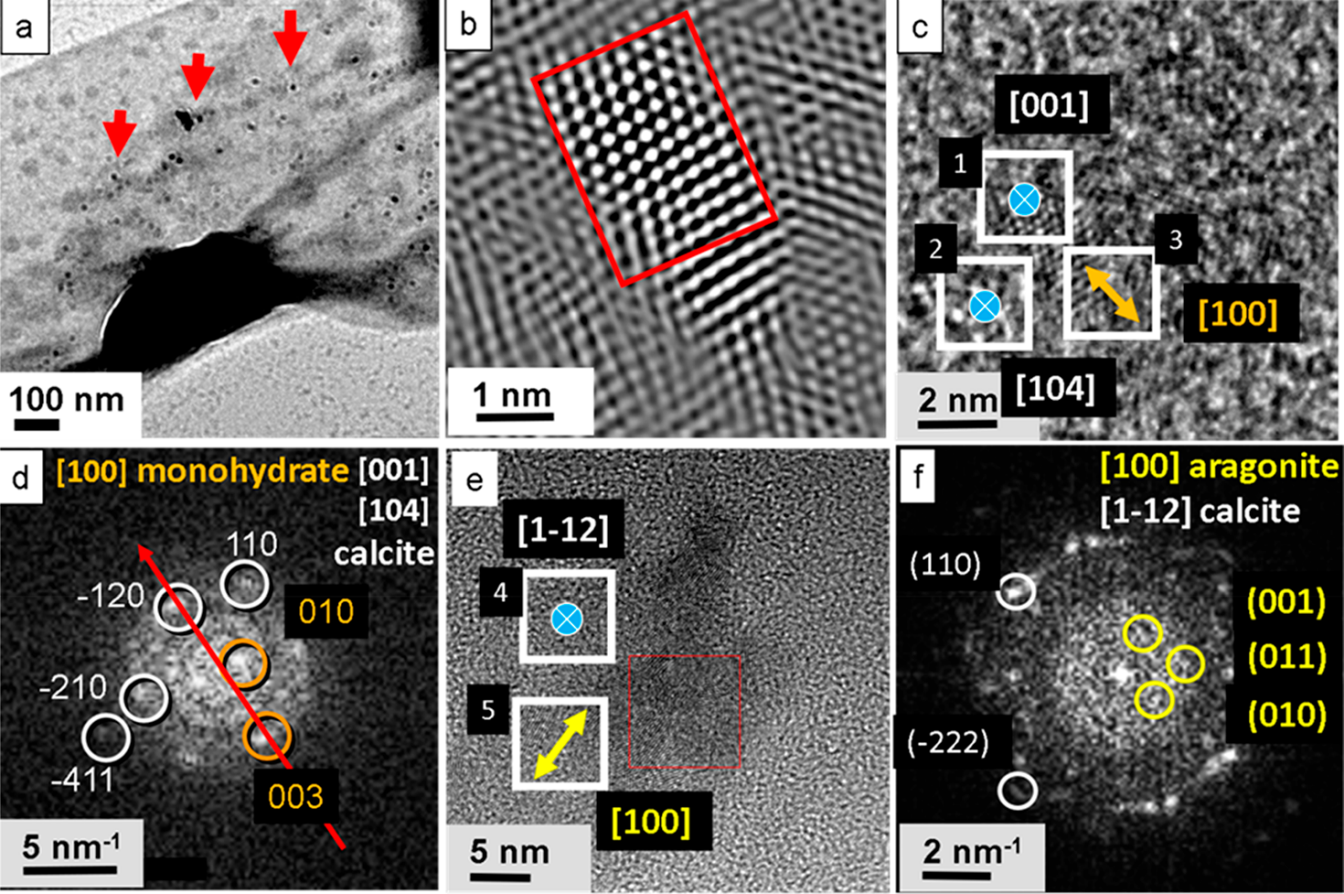
Mineralization of the whole cell. Possibly, the PG-layer at the surface is seen due to a partially discarded mineralized bacterial S-layer membrane. (a) After 6 h incubation, several nanosized calcium carbonate crystals are precipitated at the cell surface in the large pores (red arrows). (b) High-resolution TEM (Fourier filtered image) displays a calcite nanocrystal appearing as a single crystal nucleus, 3 nm in size. (c) Crystal aggregate on the bacteria surface composed of calcite nanocrystals (blue icons) together with monohydrocalcite (orange icon). (d) Corresponding FFT indicates calcite in [001] and [104] orientation and monohydrocalcite in [100] orientation. (e) Aggregate of a calcite nanocrystal on the substrate together with aragonite (yellow icon). (f) FFT indicates calcite in [11̅2] orientation and aragonite in [100] orientation.

TEM grids covered with whole cells of *G. stearothermophilus* were incubated in 10 mM CaCl_2_ solution for 6, 12, and 24 h, respectively. Afterward, the mineralization was performed by putting the activated grids on a drop of 0.1 M Na_2_CO_3_ for 1, 6, 12, and 24 h. Only a very small number of calcium carbonate crystals could be observed on the surface of the cells upon an incubation time of 6 h (Figure 4a and SI Figure S3.1). The crystals were about 3–8 nm in size. In a few cases, clusters were observed as marked by red arrows. The high-resolution image (Figure 4c) and the digitally zoomed and Fourier filtered micrograph of the same crystal (Figure 4b) show a tiny calcite single crystal with typical hexagonal symmetry and a size of about 3 nm. The measured lattice spacing amounts to about 2.49 Å corresponding to the *d*-value of the (110) reflection and thus corresponding to the [001] zone of calcite (Figure 4c,d). Also, the (4̅11) reflection was detected indicating the [104] zone of calcite. Furthermore, the [100] zone of monohydrocalcite has been observed. In another spot, the [100] zone of aragonite has been registered, together with a calcite reflex of the [11̅2] zone (Figure 4e,f). Larger crystals were polycrystalline, formed by the merging of several nanocrystals. Crystal orientations detected on the bacterial S-layer membrane are listed in Table 1.

**Table 1:** List of Orientations in Figure 4c,e Occurring on the Bacterial S-Layer Membrane

1	[001] calcite
2	[104] calcite
3	[100] monohydrocalcite
4	[11̅2] calcite
5	[100] vaterite

After 12 h of incubation time, the number of calcium carbonate crystals increased considerably. In the case of the whole bacterium, isolated fully mineralized surface layers were detected. In SI Figure S3.2a,b, the cell with the discarded and mineralized bacterial membrane is shown. The discarded bacterial membrane reveals irregularly shaped nanoporosity with pore sizes between 20 and 50 nm and larger pore aggregates (SI Figure S3.2c,d). The big pores are a hint that the outer S-layer has been discarded together with the underlying PG-layer (see also the Discussion). This kind of structuring was already observed for the native sample (Figure 2b) and uranyl-stained samples (Figure 2c,d and Figure S2.1). The abandonment of the bacterial membrane already occurred in the early stage of mineralization as indicated by SI Figure S3.2e,f. The images show a cell surrounded by the discarded membrane. After 24 h incubation, the bacterial membrane of the whole cell was completely mineralized. We often observed that the mineralized cell left behind an elongated “carpet” with a size of about 9 μm × 1.5 μm (SI Figure S3.4). The membrane shows strongly mineralized areas of about several hundreds of nanometers in diameter separated by more sparsely mineralized bright regions (SI Figure S3.5a–c). At higher magnification, a pattern within the mineralized regions resembling a fine-meshed net with ultrathin proteinaceous walls of 1 nm thickness was observed (SI Figure S3.5d,e); thus, the original S-layer structure seems to be modified. The calcite crystals grow in compartments with different sizes from 1 to 12 nm, mainly 3–5 nm (SI Figure S3.5e,f).

### II.iii Mineralized Self-Assembled S-layer

The mineralization on the reassembled and isolated S-layer took place more slowly. The first signs of mineralization became evident only after 12 h of incubation. Homogeneously distributed crystal aggregates are found over the whole sample (SI Figure S3.3a,b). The distance from aggregate to aggregate adds up to 50–100 nm whereas the sizes of the calcite clusters amount to between 5 and 50 nm (SI Figure S3.3b,c). In SI Figure S3.3d–f, mineralized aggregates are shown. In these clusters, individual calcite nanocrystals with sizes between 2 and 5 nm are present, SI Figure S3.3g,h.

**Figure 5:**
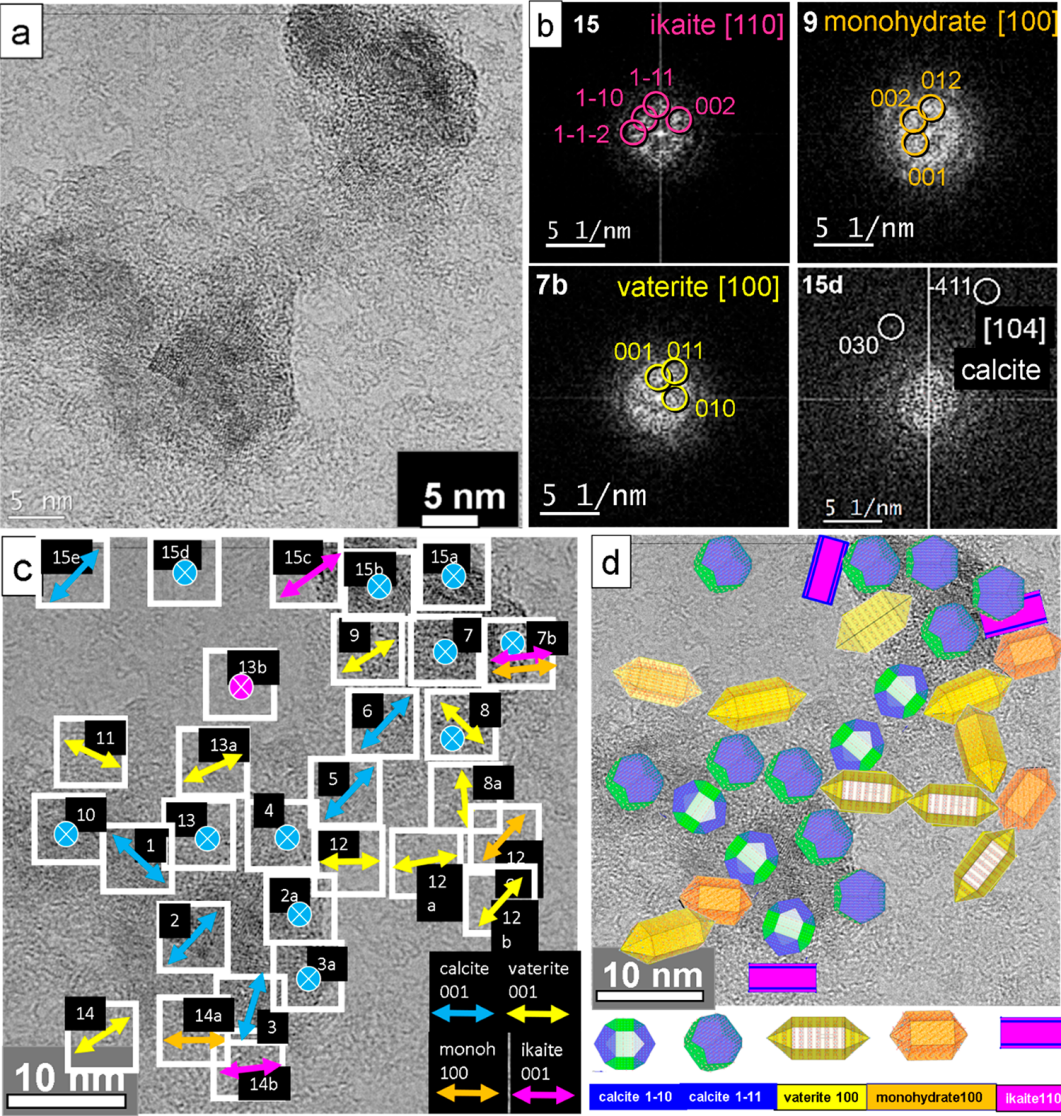
Mineralized calcium carbonate polymorphs of an S-layer of *G. stearothermophilus* after 12 h incubation. (a) High-resolution TEM image of nanocrystals on the reassembled S-layer. (b) Selected-diffraction diagrams for kinetically grown samples with favored growth directions of ikaite [110], monohydrocalcite [100], vaterite [100], and an equilibrium orientation of calcite [104]. (c) Fourier analysis of the nanocrystals indicates different in-plane orientations. The crossed insets describe the [104], [214], [001], and [11̅2] axes of calcite (in blue), and the [001] axis of ikaite (in pink), both oriented parallel to the surface normal. (d) Model derived from panel c shows nanocrystal orientations of calcite (blue), vaterite (yellow), monohydrocalcite (orange), and ikaite (pink).

In order to reveal the mineralization scenario in the small pores (3–5 nm) of a self-assembled S-layer in detail, we decided to perform an exhausting analysis as shown in Figure 5. In the high-resolution TEM image, nanocrystalline nuclei with a dimension of 2–4 nm are observed (Figure 5a). The analysis of the FFTs of the individual nanocrystals was performed, in order to derive the orientation map. In Figure 5b, selected orientations of kinetically grown (K) ikaite (110), monohydrocalcite (100), and vaterite (100) are shown, whereas the calcite (104) orientation corresponds to thermodynamic equilibrium (T) geometry (see also Discussion and SI Supplement S4).

The orientation map represents a compact image of the processes occurring during the complete growth of calcium carbonate in aqueous solution under the restrictions of nanosized compartments. In Figure 5c, the in-plane orientations of the long axis of the different polymorphs in various spots are shown. For some spots, their orientations parallel to the surface normal are indicated. The long axes of the observed calcium carbonate phases are drawn in different colors. For indexing the calcite and vaterite reflections, the models of Meyer^39^ (for calcite) and of Maslen^40^ (for vaterite with a distorted supercell; see also Demichelis et al.^41^) have been used. The *c*-axis of calcite is indicated in blue. In the case of orientations marked with a blue crossed spot, the *c*-axis corresponds to a direction out of plane. The vaterite *c*-axis orientation is shown in yellow. The monohydrocalcite *a*-axis appears in orange (indexing with data from Ehrenberger^42^). Ikaite is indicated with the orientation of the *c*-axis in pink (indexing with data from Dickens and Brown^43^). The detailed analysis of the orientation map with different calcium carbonate phases encompassing calcite, vaterite, monohydrocalcite, and ikaite will be exhaustively described in the Discussion. The complete list of orientations and phases is given in Table 2. A schematic model of the nanocrystal distribution derived from the orientation map is shown in Figure 5d.

**Table 2:** Dominating Growth Directions of the Calcium Carbonate Crystals in the Spots Shown in Figure 5c

1	(11̅0) calcite K	11	(100) vaterite K
2	(11̅0) calcite K	12	(100) vaterite K
2a	(11̅1) calcite K	12a	(100) vaterite K
	(201) vaterite K		
3	(11̅0) calcite K	12b	(100) vaterite K
3a	(214) calcite T	12c	(100) mhc K
4	(11̅1) calcite K	13	(11̅1) calcite K
5	(11̅0) calcite K	13a	(100) vaterite K
6	(11̅0) calcite K	13b	(001) ikaite T
7	(11̅2) calcite K	14	(100) vaterite K
7b	(001) calcite T	14a	(100) mhc K
7b	(100) mhc K	14b	(110) ikaite K
7b	(110) ikaite K	15a	(001) calcite T
8	(100) vaterite K	15b	(214) calcite T
	(201) ikaite K		
8a	(100) vaterite K	15c	(110) ikaite K
9	(100) vaterite K	15d	(104) calcite T
10	(11̅1) calcite K	15c	(100) calcite K

## III Discussion

The observed nanopores in the surface layer (S-layer) and in the supporting PG-layer of the cell wall are favored nucleation sites for metastable calcium carbonate polymorphs and polyamorphs. The mode of nucleation is governed by the presence of appropriate binding sites for Ca^2+^ ions as well as the geometric constraints (pore diameters).

### III.i Nucleation Sites in the S-Layer

**Figure 6:**
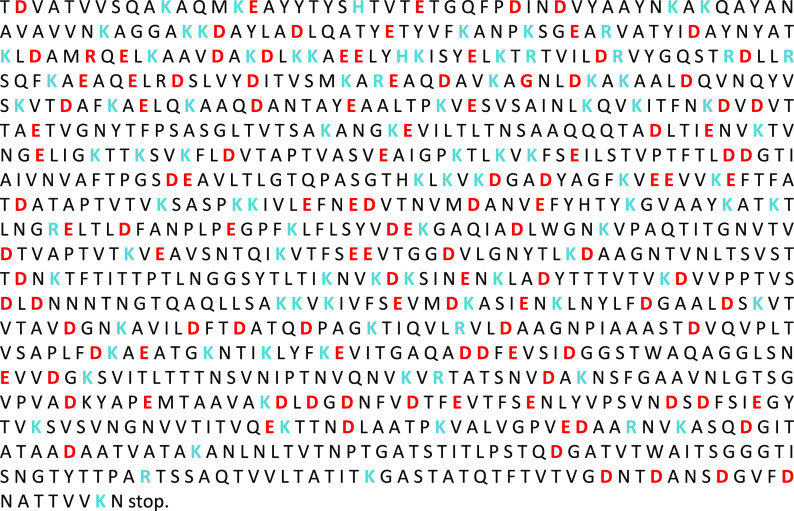
Protein sequence of S13240-ORF from *G. stearothermophilus*.^44^ The mature protein S13240 consists of 1038 amino acids. The mature protein starts with threonine (T) at position 32. The amino acids with high acidity (aspartic acid (D) and glutamic acid (E)) and high basicity (arginine (R), histidine (H), and lysine (K)) are printed in red and blue, respectively.

The mature S-layer protein S13240 consists of 1038 amino acids (see also Figure 6).^44^ The amino acids with high acidity (aspartic acid (D) and glutamic acid (E)) and high basicity (arginine (R), histidine (H), and lysine (K)) determine the folding and interactions of the proteins in the S-layer as well as the nucleation of the mineral phases. The S-layer of *G. stearothermophilus* possesses a high molar fraction of this kind of amino acid (glycine, 2.9%; threonine, 12.4%; serine, 4.8%; glutamine, 4.1%; glutamic acid, 5.3%; asparagine, 6.5%; aspartic acid, 7.6%). Thus, the presence of the strongest acidic amino acids (aspartic and glutamic acids) in the C-terminus as well as the concentration of the linking ions (e.g., calcium or magnesium) are responsible for the specific pore geometry of the S-layer. The crystalline structure of the S-layer is the result of a calcium-triggered multistep assembly pathway.^45,46^ The C-terminal crystallization leads to rigid domains which form the 2D-crystal lattice including the periodic distribution of the nanosized pores. The N-terminal crystallization yields domains binding to the negatively charged secondary cell wall polymer, which means the anchoring of the S-layer at the cell wall. As shown by Herrmann et al.,^45^ the N-terminal domains possess motional dynamics with respect to the rigid S-layer lattice-forming domains. The N-terminal domain of the mature protein shown in Figure 6 consists of the amino acids from position 32 (T) to position 270 (I) of the complete protein S13240-ORF from *G. stearothermophilus*.^44^

The C-terminal domains also are favored nucleation sites for the calcium carbonate polymorphs. The interaction of amino acids with calcite resulting in hybrid crystals was investigated by Kai et al.^47^ They assessed that noncharged polar amino acids such as, e.g., glycine and acidic amino acids are preferentially built into CaCO_3_ instead of nonpolar and basic amino acids.

Additionally, the Ca^2+^ binding positions of the S-layer proteins can act for the immobilization of other positively charged metal ions. Therefore, they can also be used not only for the removal of toxic metals from polluted waters, such as arsenic, chromate, cadmium, curium, europium, or uranium, but also for the recovery of valuable metals such as gold or platinum.^48,49^

The regulation of the S-layer structure by the presence of Ca^2+^ ions can cause an irreversible switching between a crystalline and an amorphous structure. Herrmann et al. have shown that the structure of the S-layer of *Caulobacter crescentus* changes from the two-dimensional crystalline state at higher calcium ion concentration into an amorphous aggregate state at low concentration.^50^

The observed diameter of the S-layer pores (⌀ 3–5 nm) leads to an essential restriction for the possible calcium carbonate polymorphs nucleating in these cavities. The basic structures of calcium carbonate formation in aqueous solutions are chains with a subnanometer diameter of dynamically ordered liquid-like oxyanion polymers (DOLLOPs),^41^ and so-called dense liquid phases (DLPs) with an average size around 0.9 nm.^15^ DOLLOPs and DLPs can act as prenucleation clusters. The formation of solid calcium carbonate polymorphs can occur along two alternative pathways. One follows the classical nucleation theory^15^ based on stepwise aggregation of single calcium and carbonate ions. Alternatively, there is the so-called nonclassical pathway which starts with the formation of larger thermodynamically stable liquid prenucleation clusters (PNCs) formed in the supersaturated aqueous solution.^51^ There is no phase boundary between the clusters and the surrounding solution. After further growth, they develop interfaces and become nanodroplets. These nanoscopic intermediate phases transform by concurrent accretion and dehydration into solid hydrous amorphous and crystalline polymorphs.^16,52,53^ By coalescence, solid intermediates from a few nanometers to hundreds of micrometer in size are formed.^16^

With molecular simulations and supporting experimental data, including equilibrium constants, titration curves, and X-ray absorption spectra, it has been recently shown that the classical model of nucleation of the solid phase has to be favored. By monomer addition from a solution rich in isolated ions and their pairs, the first solid phases are formed (Henzler et al.^54^).

### III.ii Influence of the Interface and Attachment Energies on the Growth Process of the Calcium Carbonate Polymorphs

The formation of crystalline calcium carbonate in a supersaturated aqueous solution of Ca^2+^ and CO_3_^2–^ ions at room temperature proceeds as a stepwise process following Ostwald’s step rule.^55^ Initially, prenucleation clusters composed of Ca^2+^ and CO_3_^2–^ ions aggregate into hydrated ikaite, monohydrocalcite (mhc), or amorphous calcium carbonate (ACC), which later can transform into the crystalline polymorphs vaterite, aragonite, or calcite depending on different pH conditions.^15,19,22,53,56−62^ Under the given growth conditions (temperature 4 °C, characteristic time scale of about 6–12 h), we can observe that metastable polymorphs grown during the kinetic stage as well as the calcite formed as the thermodynamic equilibrium phase should be observed. Phenomenologically, the growth rate and the relative stability of the various polymorphs can be characterized by the surface energies of the different crystal faces and their attachment energies (de Leeuw and Parker 1998).^63^ The attachment energy is defined as the negative value of the released energy per molecule when a slice of thickness *d*_*hkl*_ crystallizes onto a crystal face (*hkl*). Surfaces with high values of the surface energy and high absolute values of the (negative) attachment energy grow out with a high growth rate, whereas the surfaces with low values are expressed as large faces in the equilibrium morphology. In Table 3, few examples for values of surface and attachment energies for the crystalline phases observed in the presented experiments are summarized. These data have been selected from a theoretical study on nanoscale morphology and surface stability, given by Sekkal and Zaoui in 2013.^64^ In particular, the results based on a force field model for the interatomic interactions developed by Xiao et al. in 2011^65^ have been presented. This model also reproduces experimental data for other structural and thermodynamic properties of CaCO_3_ well.

**Table 3:** Selected Values for Surface Energies and Attachment Energies for Crystalline Calcium Carbonate Polymorphs^64^

	surface energy (J/m^2^)	attachment energy (eV)
ikaite	(100) 0.52	(100) −8.19
	(010) 0.37	(010) −5.79
	(001) 0.20	(001) −2.57
mhc	(100) 1.54	(100) −7.82
	(010) 1.21	(010) −6.62
	(001) 0.99	(001) −5.66
vaterite	(100)CO_3_ 1.54	(100)CO_3_ −91.41
	(110)Ca 0.87	(110)Ca −107.35
	(111)Ca 0.78	(111)Ca −109.70
calcite	(104) 0.51	(104) −8.69
	(100) 0.64	(100) −14.37
	(110) 0.76	(110) −24.58
	(012) 1.56	(012) −100.78

The orientations of the surfaces showing in the dominating growth direction of the various polymorphs are summarized in Table 2. The main part of the crystals is grown in a kinetic regime (K). In Figure 5b, few examples are selected for kinetically grown ikaite (with favored growth direction [110]), monohydrocalcite (with favored growth direction [100]), vaterite (with favored growth direction [100]), and the equilibrium face orientation [104] of calcite. The complete set of the selected-diffraction diagrams of the spots shown in Figure 5c is given in the Supporting Information, Supplement S4. Only in the spots 15d with calcite (104), 3a and 15b with calcite (214), 7b and 15a with calcite (001), and 13b with ikaite (001) the morphology has approached a constrained thermodynamic equilibrium (T). The faces with calcite (104) and with ikaite (001) are faces of minimum surface energies. The normal of the face with calcite (001) deviates by 4.18°, and the normal of the face with calcite (214) deviates by 8.25° from the normal of the equilibrium face (104) of calcite.

### III.iii Ikaite Formation in the S-Layer

By computer simulations combined with the analysis of experimental data, Demichelis^41^ and co-workers have shown that polymeric chains of Ca and carbonate ions termed dynamically ordered liquidlike oxyanion polymers (DOLLOPs) are an initial form of prenucleation structures. In aqueous solution, calcium carbonate exists as a nonpolar structure of dipole arrays 8-fold coordinated by water with the mean Ca–O distance of 2.46 Å.^66^ For the 8-fold coordinated calcium carbonate in ikaite, a mean Ca–O distance of 2.469 Å at 243 K has been found.^67^ Therefore, it has been proposed by Chaka that at low temperature small ikaite-like prenucleation clusters crystallize out of solution into ikaite.^62^ The ikaite nanocrystals are observed as metastable solid precipitations in the S-layer pores after 12 h (see the spots 7b, 13b, 14b, and 15c in Figure 5c and Table 2).

### III.iv Kinetically Controlled Solid-State Transformation of Ikaite

#### Vaterite Growth by Solid-State Transformation

The vaterite spots 8, 8a, 9, 13a, and 14 with the face orientation (100) and the [001] vector in this lattice plane can be explained as the solid-state transformation of ikaite nanorods. As described by Tang et al. in 2009,^57^ by dehydration of the ikaite lattice and translations of the CO_3_^–^ ions, a distorted vaterite lattice symmetry^68^ is formed. The orthorhombic ikaite lattice is oriented with its [001] and [010] axes along the [001̅] and [010] axes of vaterite, respectively. Caused by the already predefined favored [100] growth directions of the ikaite crystals with minimum attachment energy *E*_att_ = −8.91 eV, they are transformed by dehydration into vaterite nanocrystals growing in the [100] direction with the small attachment energy of −91.41 eV for the (100) face (Table 3).

#### Monohydrocalcite Growth by Solid-State Transformation

Together with ikaite, in addition, few monohydrocalcite (mhc) spots are observed among the precipitates on the S-layer after 12 h (spots 12c and 14a). Both precipitation reactions are exothermic even at temperatures up to 373 K.^62^ As can be seen in the spots 7b, 12c, 13b, 14a, 14b, 15c of Figure 5b, the nanosized constraints stabilize ikaite and monohydrocalcite at 4 °C. The spots 7b, 14a, 14b, and 15c are metastable kinetic structures of ikaite and monohydrocalcite, respectively. As for vaterite, [100] is the favored growth direction of mhc. The mobility of ions in the mhc lattice is limited in comparison to that in the ikaite lattice (with a higher content of water leading to interconnected columns of water molecules in the ikaite lattice^69^). Thus, large crystalline structures of anhydrous carbonate phases are formed only by a dissolution/precipitation process from mhc. However, in nanosized mhc crystals, such a limited mobility should not be relevant in structural transformation due to the short distances in the nanocrystals. Therefore, we assume that mhc could be an intermediate phase of the dehydration reaction: ikaite → mhc → vaterite.

### III.v Precipitation from Hydrated ACC

Recently, Demichelis et al.^41^ have shown that calcium and carbonate ions rapidly aggregate in solution to form stable filamentous clusters. These precursors have an unusual and very dynamic structure consisting of chains of alternating cations and anions. This new type of species has been termed a “dynamically ordered liquid-like oxyanion polymer” (DOLLOP). It represents the structural form of prenucleation clusters. Similar structures, so-called dense liquid phases (DLPs), have been observed by Smeets et al.^15^ by combined experimental and computational investigations of the precipitation of CaCO_3_ in dilute aqueous solution. Near the critical temperature, DLP clusters composed of 4–7 H_2_O per CaCO_3_ are formed by phase separation, with the average coordination in the largest clusters (average size around 0.9 nm) of around 2.8. For comparison, in amorphous calcium carbonate the water content is significantly lower (about 1.4 H_2_O per CaCO_3_) than in DLP. In the first solid structures grown by aggregation of these DLPs, a corrugated spherical morphology characteristic of vaterite has been detected by SEM studies. In this connection, the studies of Gebauer et al.^53^ and Cartwright et al.^16^ are of relevance. They show different amorphous calcium carbonate (ACC) phases with distinct shorter-range orders, called polyamorphs. In additive-free ACCs precipitated from equilibrated, slightly supersaturated (metastable) solutions of calcium carbonate, short-range structures related to calcite and vaterite, respectively, have been detected.^53^ The authors assume that the mentioned formation of chain-like and highly dynamic structures in prenucleation clusters may be the basic principle behind protocrystalline structuring in intermediate ACC clusters. The structuring depends on the pH value, pH 8.75 and pH 9.80, respectively. In the present study, the experiments have been conducted at pH 7.

In comparison to ikaite, ACCs need larger pores due to the smaller packing density of the lattice. Hydrated ACCs can grow only in confinements with a size of at least about 10 nm as observed by Stephens et al.^70^ in a biomimetic experiment with crossed cylinders for the nucleation containment. In larger containments, monohydrocalcite is precipitated from hydrated ACC.^71^ Amorphous calcium carbonate (ACC·*x*H_2_O) can be observed in a hydrated form with *x* typically 0.5 to 1.4 mol water per mole CaCO_3_. In biogenic structures of ACC, the stoichiometry of CaCO_3_·H_2_O is found.^23,24^ After dehydration in air, ACC crystallizes. Following the thermal stability pathway, *anhydrous* ACC transforms as *anhydrous* ACC → vaterite → aragonite → calcite.^58^ Alternatively, *hydrous* ACC can be transformed via monohydrocalcite^72^ into anhydrous crystalline polymorphs.^59,73^ Zhuo et al. have shown that the size of the ACC clusters determines the polymorph selection in solution.^74^ For ACC nanoparticles with an average size ranging from ∼66 to ∼196 nm, the portion of vaterite to calcite increased with decreasing particle size. The observed significant influence of the pore size on the starting point of the Ostwald’s step cascade explains the observed polymorphs grown in the PG-layer.

### III.vi Nucleation Sites in the PG-Layer

**Figure 7:**
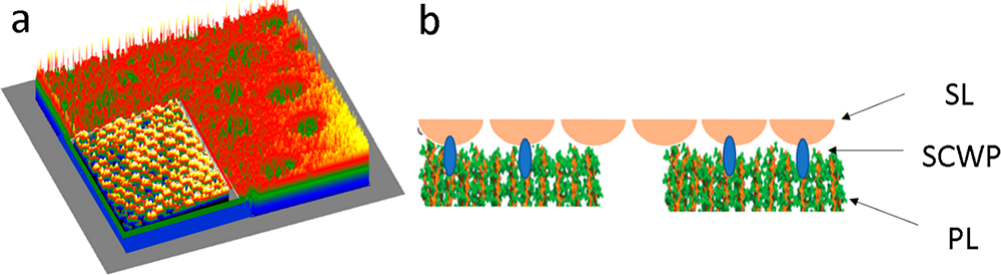
Main components of the membrane of Gram-positive bacteria. (a) Schematic representation of underlying PG-layer (orange/green) together with the clamped upper S-layer (yellow inset bottom left) covering the surface derived from the AFM and TEM images in Figure 2. (b) Schematic of the link structure and nanopore distribution in the membrane: the surface protein layer (SL) is linked with the peptidoglycan layer (PL) by secondary cell wall proteins (SCWPs); there are two families of nanopores, small pores (⌀ 3–5 nm) of the S-layer and larger pores of the PG-layer (⌀ 20–50 nm).

The N-terminal part of the S-layer protein of *G. stearothermophilus* is covalently linked to the PG backbones by so-called secondary cell wall polymers (SCWPs). Specific S-layer homologue (SLH) motifs are involved in these bonds (Figure 7). Additionally, there are further direct links between the S-layer and the PG-network.^6,34,75−77^ The large pores (⌀ 20–50 nm) of the PG-layer shown in Figure 2c,d,g,h are possible nucleation sites for calcium carbonate. Similar large pores (up to 60 nm) have been observed recently in the PG-layer of *Bacillus subtilis* by Pasquina-Lemonche et al.^78^

The FFT data in Figure 4d from a partially mineralized discarded bacterial membrane show that in such a large pore monohydrocalcite is grown preferentially in orientation [100]. For monohydrocalcite, the corresponding attachment energy is −7.82 eV^65^ (see Table 3). These results show that the [100] surface is less stable than the other two faces. The FFT data in Figure 4f show that aragonite is also stabilized in these pores. The aragonite face is also oriented in [100]. That corresponds to a less stable face due to attachment energy −10.75 eV.^65^ It means that monohydrocalcite and aragonite are kinetically grown structures in the large pores of the PG-layer. The detected calcite spots in Figure 4c–f show that in a following transformation equilibrium faces with orientation (104)^65^ or near-equilibrium orientations (001) and (11̅2) are formed.

**Figure 8:**
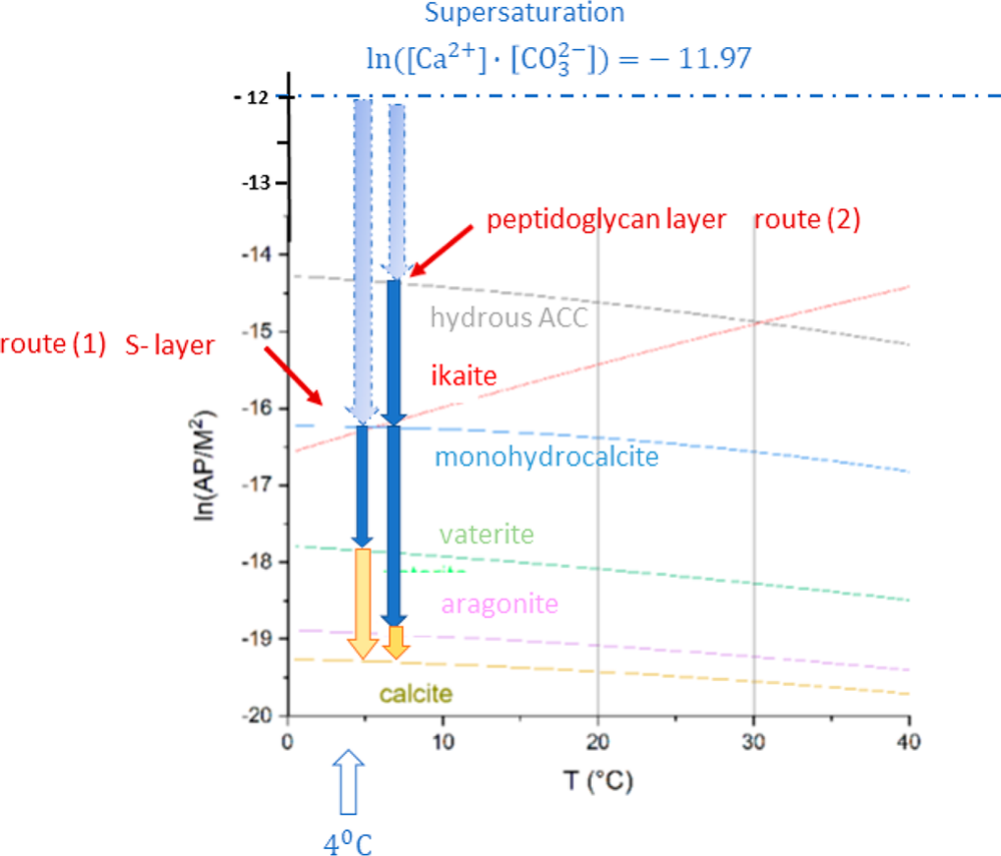
Precipitation diagram of the calcium carbonate polymorphs for the replacement reaction of calcium chloride with sodium bicarbonate in the presence of the S-layer as well as the peptidoglycan-layer of *G. stearothermophilus*. In route 1, the geometric containment (pore diameter of about 5 nm) causes the ikaite precipitation as the first solid phase, whereas at route 2 (pore size larger 25 nm) hydrous amorphous calcium carbonate (ACC·*x*H_2_O with *x* ≈ 1) is grown as the first solid phase. In route 1, after the solid-state transformation of ikaite into vaterite, a dissolution/precipitation reaction yields the thermodynamically stable calcite. In route 2, the hydrous ACC is transformed by solid-state transformations via monohydrocalcite into aragonite. Finally, a dissolution/precipitation reaction leads to calcite.

In all of the spots of the mineralized self-assembled S-layer (with its small pores), aragonite could not be detected. This means that the metastable aragonite is an intermediate reaction product of the initially precipitated ACC in the larger pores. This result corresponds to the biomimetic formation of aragonite nanorods in nanoscale pores (25 nm) in the absence of any additives published by Zeng et al.^38^ Thus, the larger pores in the PG-layer offer a second route for the formation of metastable polymorphs (see also Figure 8).

### III.vii Thermodynamically Controlled Phase Formation of Calcite

#### Calcite Growth by a Dissolution/Precipitation Mechanism

The simulation shows that in the thermodynamic equilibrium the hydrated neutral (104) surface of calcite is the most stable surface. It is observed in spot 15d in Figure 5b. At this surface, calcium as well as carbonate ions exist in an arrangement, which yields a neutral plane. As shown by Gal et al.,^79^ the preferred growth of this surface can be explained by accretion of ACC nanoparticles concomitant with ion-by-ion growth. The spherical ACC particles are transformed into faceted particles by local dissolution–reprecipitation. By surface diffusion, the particles migrate afterward to kinks or steps at the {104} face. The simulation of Sekkal and Zaoui^64^ yields 0.51 J m^–2^ for its surface energy, which is the minimum value among all possible orientations of hydrated calcite surfaces. There are two similar surface orientations of the calcite (001) in spots 7b and 15a, and (214) in spots 3a and 15b which differ from the (104) orientation only by θ_(001)_ = 4.18° and θ_(214)_ = 8.25°, respectively. Thus, these surfaces can be considered as close to thermodynamic equilibrium. All the other calcite surfaces seem to be formed in a kinetic growth regime. For example, the simulation shows that the attachment energies have low values at the surfaces (100) with *E*_att_ = −14.37 eV, (110) with *E*_att_ = −24.58 eV, and (012) with *E*_att_= −100.78 eV. The dominating source for the formation of these calcite nanocrystals is the dissolution reaction of vaterite.

#### Calcite Growth on the S-Layer Template or Epitaxially on Vaterite and Monohydrocalcite Particles

**Figure 9:**
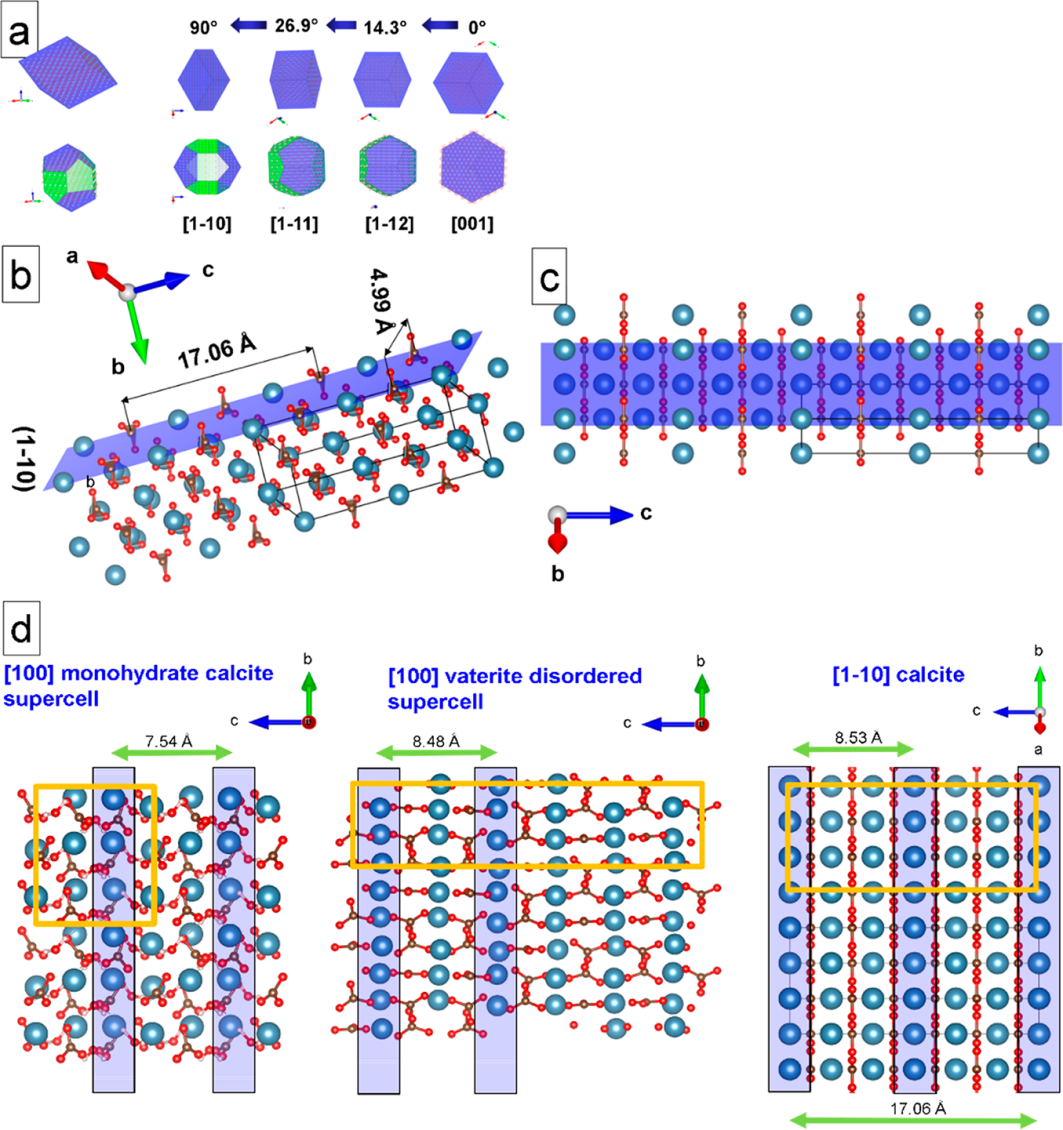
Calcite and precursor CaCO_3_ polymorphs growth on the S-layer. (a) Models of calcite nanocrystal orientations found in the image. Top: Rhombohedral calcite habit. Numbers between the arrows are indicating angles between the different orientations. Bottom: Calcite crystal habit with an additional set of prismatic facets {11̅0}. (b, c) (11̅0) surface of calcite is shown, which is in contact with the bacterial S-layer as well as with a vaterite particle. (d) Comparison of surfaces and summary of possible epitaxial relations between monohydrocalcite, vaterite, and calcite. Specific columns of Ca atoms along the *b*-axis are highlighted in blue. These Ca atoms are lying on the same upper level having a lateral distance of 7.54–8.52 Å. The Ca atoms between the columns (not highlighted) are positioned below this surface (2.5 Å or lower). Unit cells have been indicated in orange.

The {11̅0} planes of the 3–5 nm sized calcite crystals in spots 1, 2, 3, 5, and 6 are either in contact with the biological substrate retracing the topology of the protein surface layer (Figure 9a–c) or could be also epitaxially grown on vaterite or monohydrocalcite particles (see Figure 9d). The [100] surfaces of the two metastable polymorphs shown in Figure 9d, left and center, can act as substrates for epitaxial growth of calcite in [11̅0] orientation (see Figure 9d, right). Calcite nanocrystals are visualized in Figure 9a for different crystal habits. At the top, the typical rhombohedral shape is sketched, and in the row additionally a set of prismatic facets {11̅0} is shown. The schematic orientation map of the CaCO_3_ nanocrystals on the S-layer is derived from the high-resolution micrograph shown in Figure 5a.

### III.viii Mineralization Driven Evolution of the Bacterial Membrane

The TEM images in Figure 4 and SI Figure S3.1 show that after 6 h incubation the mineralized bacterial membrane is already partially discarded from the cell wall. The image of the not fully mineralized membrane reveals that the membrane is already dropped at the beginning of the mineralization process. It means that the cell possesses an effective protection mechanism to avoid a complete coating with a mineral layer. It is known that bacteria can grow a new S-layer when for some reason a former S-layer has been discarded. A rough estimate yields that about 500,000 S-layer monomers are necessary for completely covering a whole rod-shaped bacterium.^80,81^ The transport to the cell wall and the secretion of the protein across the cell envelope are the essential processes governing the time required for the S-layer formation. It is assumed that typically 100–500 copies of a single polypeptide chain per second can be synthesized during exponential growth inside the cell.^80,82^ Such estimates lead to a growth time for a new S-layer of about 20 min. That allows the conclusion that already first nucleation events of mineral particles seem to initiate the separation of the disturbed bacterial membrane in order to secrete a new structure equipped with full biological functionality. There are two structural features which contribute to this behavior: the stabilization of metastable polymorphs of calcium carbonate by the confinement in the S-layer, and the concurrent repeated discarding of the partially mineralized bacterial membrane with the secretion of new bacterial membranes. If we include the PG-layer into the consideration, then we can decide a 2-fold optimization of the cell wall: the stabilization against critical mechanical load caused by the layered structure with different mechanical properties, and the delayed formation of the precipitation of a stable calcite coating by the favored formation of metastable calcium carbonate polymorphs or polyamorphs.

## IV Conclusions

The mechanism of calcium carbonate deposition at the bacterial surface membrane of *G. stearothermophilus* has been disclosed at the atomic scale by high-resolution TEM and complementary AFM studies of the membrane structure. After an induction time larger than 6 h, amorphous calcium carbonate is not relevant as the intermediate phase for the mineralization of the S-layers. The small pores with sizes of 3–5 nm in the S-layers induce a preferred incorporation of stable liquid prenucleation clusters which in the following process step immediately crystallize into ikaite. Also, the second hydrated crystalline polymorph monohydrocalcite is stabilized by the narrow biomolecular confinement due to kinetic effects. These hydrated metastable crystalline phases afterward transform into the anhydrous vaterite by a solid-state transformation or into thermodynamically stable calcite by a dissolution–precipitation reaction in the aqueous solution. In the larger pores with sizes of 20–50 nm in the PG-layer, hydrated ACC nucleates followed by a transformation into monohydrocalcite and aragonite, and the precipitation of calcite. With a hydrated neutral (104) surface, calcite is grown on top of the S-layer, or heteroepitaxially at vaterite nanoparticles and monohydrocalcite in the [11̅0] zone orientation.

The nested precipitation of metastable calcium carbonate polymorphs and polyamorphs in the structured nanopores cell membrane is the key mechanism for the biomineralization under extreme environmental conditions. Probably, it could also be a more general mechanism for other Gram-positive bacteria with an S-layer. The first nucleation events of mineral particles initiate the separation of the disturbed S-layer membrane from the cell wall in order to secrete a new membrane equipped with full biological functionality. This behavior reflects the essential feature of forced biomineralization: the survival strategies of prokaryotes and eukaryotes using protective advantages of biomineralization due to functionalization of their cell envelopes^1^ in the nested formation of calcium carbonate polymorphs in the bacterial surface membrane of *G. stearothermophilus* DSM 13240.

## V Experimental Section

### V.i Cultivation of the Cells and Protein Isolation

We used the S-layer forming microbial strain *G. stearothermophilus* DSM 13240. The cultivation was performed in a lysogeny broth (+) medium (0.5 wt % yeast extract, 0.5 wt % NaCl, 2 wt % peptone at pH 7.4) at 67 °C and 200 rpm in a warm air shaker. For production of authentic S-layer protein S13240, the strain was cultivated in 1 L of prewarmed medium overnight. After harvesting of the cells at 3500 rpm at 4 °C, the pellet was washed 3 times with Tris buffer (10 mM Tris/HCl pH 7.5). In order to generate a reversible denaturation of the authentic S-layer proteins, the pellet was resuspended in 10 volumes of 2 M guanidine hydrochloride and occasionally slightly shaken for 30 min at room temperature. Subsequently, the cells were pelleted at 3500 rpm and 10 °C for 15 min, and the S-layer containing the supernatant was dialyzed against distilled H_2_O at 4 °C under constant stirring overnight. In order to investigate the mineralization of the whole cells, the *G. stearothermophilus* DSM 13240 cells were harvested at 3500 rpm and washed once with distilled H_2_O. Subsequently, the cells were resuspended in double distilled H_2_O and an optical density of OD_578_ 1 was adjusted (corresponds to about 10^9^ cells/mL).

### V.ii Atomic Force Microscopy

AFM imaging was done with an Asylum Research Cypher atomic force microscope (Asylum Research Santa Barbara, CA, USA). Measurements were performed in liquid using the AC mode. A cantilever “BioLever mini” (Olympus BL-AC40TS-C2) with a resonance frequency of about 25 kHz in water and a stiffness of 0.09 N m^–1^ has been used. A 10 μL portion of recrystallized S-layer sheets was dropped on freshly cleaved mica attached to a steel sample puck. They were left undisturbed for 5 min to promote adhesion. Most of the supernatant was removed with a pipet and replaced by a droplet of recrystallization buffer (1.5 mM Tris, 10 mM CaCl_2_, pH 8) to keep the sample in liquid upon transfer to a sample stage on AFM and following the measurement. The temperature was kept at 25 °C, scan frequency at 0.5 Hz, and scan angle at 90°.

### V.iii Transmission Electron Microscopy

The TEM experiments were carried out at the Special Laboratory Triebenberg for Electron Holography and High-Resolution Microscopy at the Technische Universität Dresden. A field emission microscope CM 200 FEG/ST-Lorentz (FEI company, Eindhoven, NL) equipped with a Gatan 1 × 1 k slow-scan CCD camera was used to perform the high-resolution TEM investigations of the materials. The analyses of the TEM images were realized by means of the Digital Micrograph software (Gatan, USA).

### V.iv Coating of TEM Grids

Reassembly of the isolated S-layer proteins (1 mg/mL) was carried out for ca. 20 min at room temperature. The carbon-coated Cu-grids were put on the surface of an S-layer assembly and monomer containing protein solution of S13240. TEM grids were put on the surface of a water solution with resuspended cells also for 20 min at room temperature in order to coat them with whole cells. Thereafter, the excess proteins and cells were removed by washing with double distilled H_2_O.

### V.v Mineralization

Mineralization of nonstained (native) reassembled proteins or whole cells on carbon coated TEM grids was performed by placing the grids on a drop of 10 mM CaCl_2_ solution for 6, 12, and 24 h, respectively. The incubation took place at room temperature in the first hour and at 4 °C afterward. Excess salt solution was removed by putting two times the number of grids on a drop of double distilled water for 30 s. In order to generate CaCO_3_, the grids were placed for 6, 12, and 24 h on a drop of 0.1 M Na_2_CO_3_. To remove unbound CaCO_3_, after the reaction, the grids were placed twice for 30 s on the surface of a drop of double distilled water. Subsequently, the grids were investigated by TEM.

## Supporting Information Available

Supplemental materials and additional figures regarding supersaturation of the reaction, TEM images, incubation time series, and Fourier analysis (PDF)
