# Supplementary material for: Nested Formation of Calcium Carbonate Polymorphs in a Bacterial Surface Membrane with a Graded Nanoconfinement: An Evolutionary Strategy to Ensure Bacterial Survival
Source: ACS Biomater Sci Eng. 2022 Jan 7;8(2):526–39. doi: 10.1021/acsbiomaterials.1c01280 (PMC8848282; doi:10.1021/acsbiomaterials.1c01280)
Supplement: Supplementary file 1 — ab1c01280_si_001.pdf [file ab1c01280_si_001.pdf]

Supplemental Information for

"Nested Formation of Calcium Carbonate Polymorphs in a Bacterial Surface Membrane with a graded Nano confinement - An Evolutionary Strategy to Ensure Bacterial Survival?!"

Paul Simon[1*], Wolfgang Pompe[2‡], Denise Gruner[3†], Elena Sturm[1#], Kai Ostermann[3], Sabine Matys[4], Manja Vogel[4], Gerhard Rödel[3]

[1]Max Planck Institute for Chemical Physics of Solids, Nöthnitzer Straße 40, 01187 Dresden, Germany

[2]Technische Universität Dresden, Institute of Materials Science, Helmholtzstr. 7, 01069 Dresden, Germany

[3]Technische Universität Dresden, Institute of Genetics, Zellescher Weg 20b, 01217 Dresden, Germany

[4]Helmholtz-Zentrum Dresden-Rossendorf, Helmholtz Institute Freiberg for Resource Technology Bautzener Landstraße 400, 01328 Dresden, Germany

[†] Technische Universität Dresden, Polymeric Microsystems, Helmholtzstr. 100, 01069 Dresden, Germany

[‡] Buchenweg 15, 01737 Tharandt, Germany

[#]University of Konstanz, Physical Chemistry, POB 714, D-78457 Konstanz, Germany

Supplement 1: Supersaturation of the calcium chloride/ sodium carbonate replacement reaction.

In order to calculate the activity product AP of the calcium and carbonate ions of a solution of 10 mM $CaCl_2$ and 0.1 M Na_2CO_3 in distilled water, we consider the simplified system of an ideal solution substituting the ion activities by ion concentrations with

$$\mathrm{AP} = [\mathrm{Ca}^{2+}] \cdot [\mathrm{CO}_3^{2-}] \quad (1)$$

For the dissociation equilibrium of calcium chloride, we use data derived by Saeed et al.[1]

with $$\mathrm{K}_{\mathrm{CaCl}_2} = 2.25 \cdot 10^{-2} \,. \quad (2)$$

Thus, we get for the reaction in the equilibrium

$$\mathrm{CaCl}_2 \leftrightarrow \mathrm{Ca}^{2+} + 2\mathrm{Cl}^{+} \quad (3)$$

$$\mathrm{K}_{\mathrm{CaCl}_2} = \frac{[\mathrm{Ca}^{2+}]\cdot[\mathrm{Cl}^-]^2}{[\mathrm{CaCl}_2]} \quad (4)$$

$$[\mathrm{Ca}^{2+}] = \frac{1}{2}.\,[\mathrm{Cl}^-] \quad (5)$$

$$[\mathrm{Ca}^{2+}] = \frac{1}{2}\cdot(0.45)^{\frac{1}{3}} \cdot 10^{-1}\mathrm{M} = 0.038\ \mathrm{M} \quad . \quad (6)$$

Sodium carbonate is very soluble in water. It dissociates into sodium ions $\mathrm{Na}^+$and carbonate CO_3^{2-}ions which form bicarbonate HCO_3^- ions with the water.[2] Based on data from Nakayama[3] we cut down the description of the dissociation equilibrium to

$$\mathrm{NaHCO}_3 \leftrightarrow \mathrm{Na}^+ + \mathrm{H}^+ + \mathrm{CO}_3^{2-} \quad (7)$$

$$\mathrm{K}_{\mathrm{NaHCO}_3} = \frac{[\mathrm{Na}^+]\cdot[\mathrm{H}^+]\cdot[\mathrm{CO}_3^{2-}]}{[\mathrm{NaHCO}_3]} \quad . \quad (8)$$

With $\mathrm{K}_{\mathrm{NaHCO}_3} = 4.68 \cdot 10^{-11}$ (Nakayama 1970) we get for a drop of 0.1 M $NaHCO_3$

$$[\mathrm{CO}_3^{2-}] = 1.67 \cdot 10^{-4}\ \mathrm{M} \quad (9)$$

and for the activity product

$$\ln\left([\mathrm{Ca}^{2+}] \cdot [\mathrm{CO}_3^{2-}]\right) = -\,11.97 \quad . \quad (10)$$

Supplement S2: TEM images of the S-Layer of *G. stearothermophilus* DSM 13240

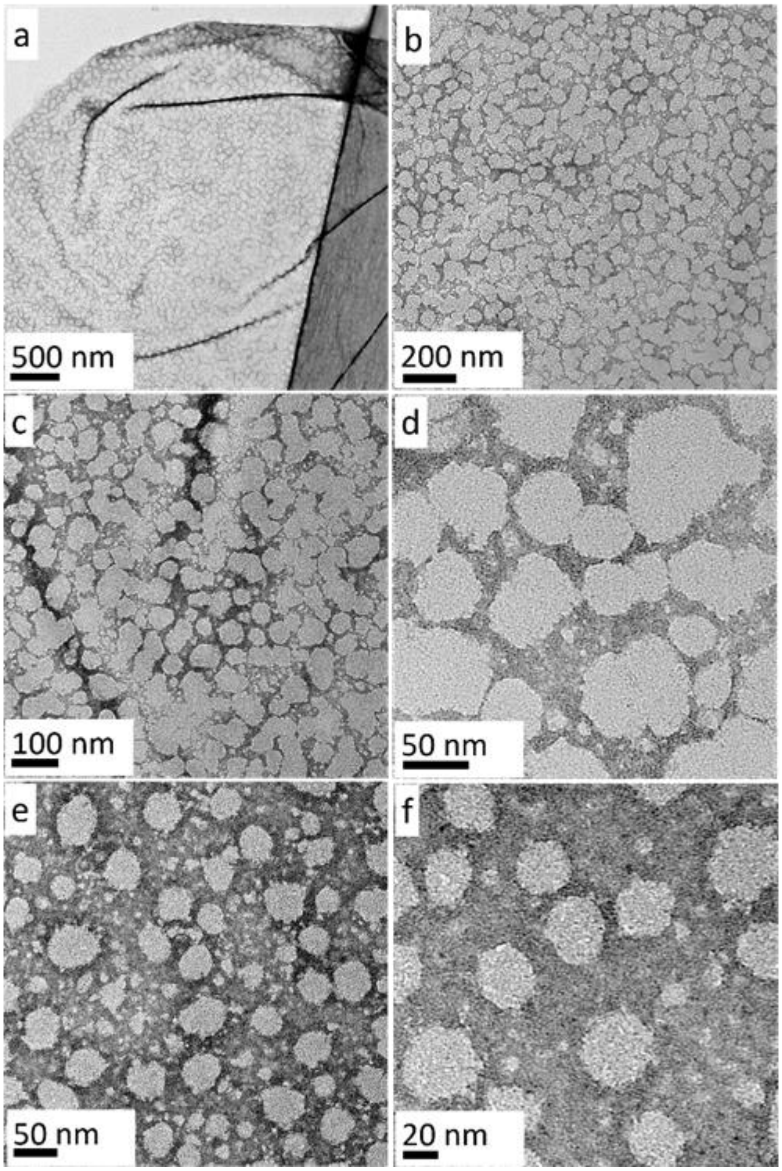

Figure S2.1. TEM micrographs of (uranyl stained) isolated, exfoliated bacterial membrane without *G. stearothermophilus* cell. (a) Overview TEM image. (b) Zoom into region of interest. (c) Further enlargement reveals pore sizes up to 100 nm. In some cases, they fuse to larger pores up to 200 nm size. (d) The image shows pores in the size range of about 20-50 nm. Additionally, at higher magnification, nanopores with sizes predominantly from 3 - 5 nm can be resolved. (e) The membrane shows also parts where the pores are less densely distributed. (f) Magnified image of (e) displays additional nanopores irregularly distributed between the larger pores.

Supplement S3: Time series for the incubation of the *G. stearothermophilus* cell with $CaCl_2$ and Na_2CO_3.

The incubation times 6, 12, 24 hours for Na_2CO_3 solution were the same as for the $CaCl_2$ solution. After washing the grid, the $CaCO_3$ crystals were investigated by TEM. Only a very small number of calcium carbonate crystals could be observed on the surface of the cells upon an incubation time of 6 hours (Figure 4a and Figure S3.1). The crystals were about 3-8 nm in size. In few cases they were clustered as marked by red arrows in Figure S3.1b. The high-resolution image (Figure S3.1c) and the digitally zoomed and Fourier filtered micrograph of the same crystal (Figure S3.1d) show a tiny calcite single crystal with typical hexagonal habit and a size of about 3 nm. The measured lattice spacing amounts to about 2.49 Å corresponding to the d-value of the (110) lattice plane. (Figure S3.1e): The fast Fourier transform shows (110), (-120) and (-2-10) lattice planes of calcite [001] zone. Also, -411 reflection was detected indicating the [104] zone of calcite and two reflexes of the [100] zone of monohydrocalcite. In Figure S3.1f the idealized model of calcite single nanocrystal morphology grown in [001] and [104] direction, and of the [100] oriented monohydrocalcite on the protein substrate is displayed. Larger crystals were polycrystalline formed by merging of several nanocrystals.

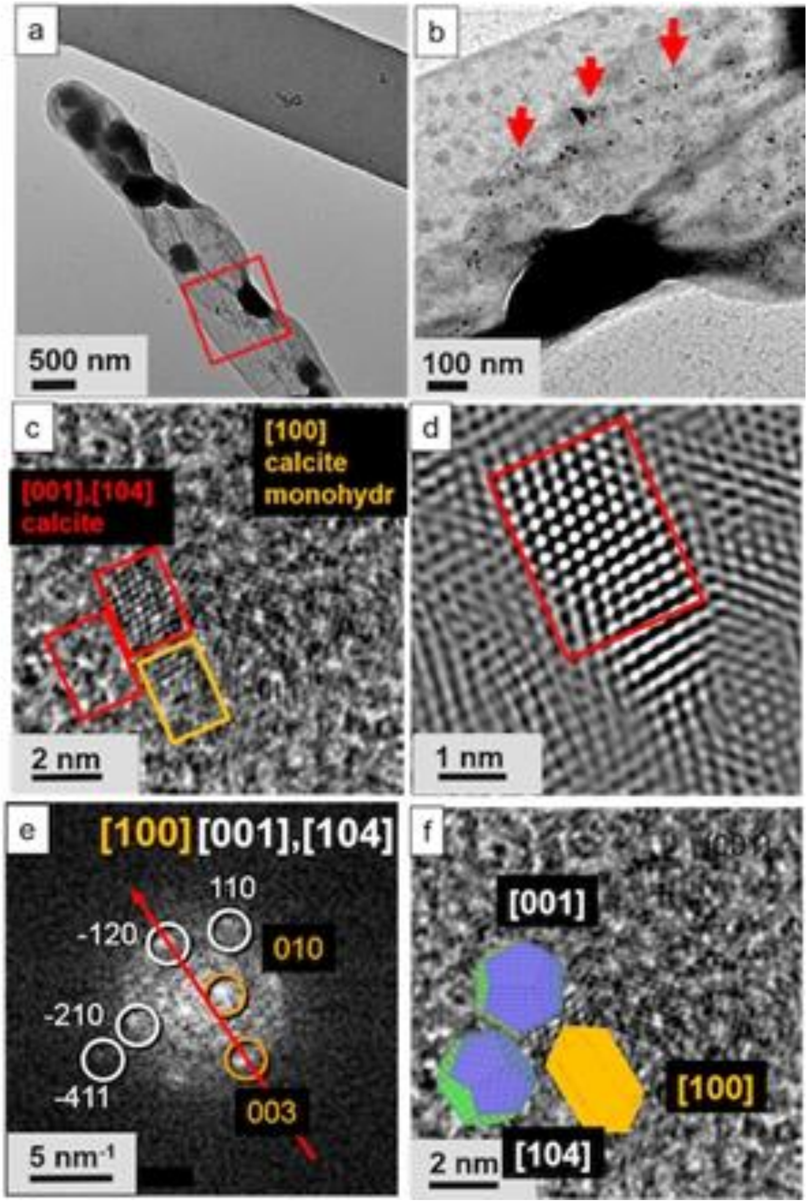

Figure S3.1. 6 hours incubation. (a) Overview TEM image of first mineralization step of the whole cells of *G. stearothermophilus*. (b) Zoomed-in image taken from red frame in (a). After 6 hours incubation several nanosized calcium carbonate crystals are deposited (red arrows). (c) High-resolution TEM displays a calcite nanocrystal appearing as a single crystal nucleus with 3 nm in size. (d) Fourier filtered image. (e) The fast Fourier transform shows (110), (-120) and (-2-10) lattice planes of calcite [001] zone. Also, -411 reflection was detected indicating the [104] zone of calcite and two reflexes of the [100] zone of monohydrocalcite. (f) Model of calcite nanocrystals with equilibrium faces of [001] and [104] orientation grown on the bacterial membrane, together with monohydrocalcite grown in [100] direction.

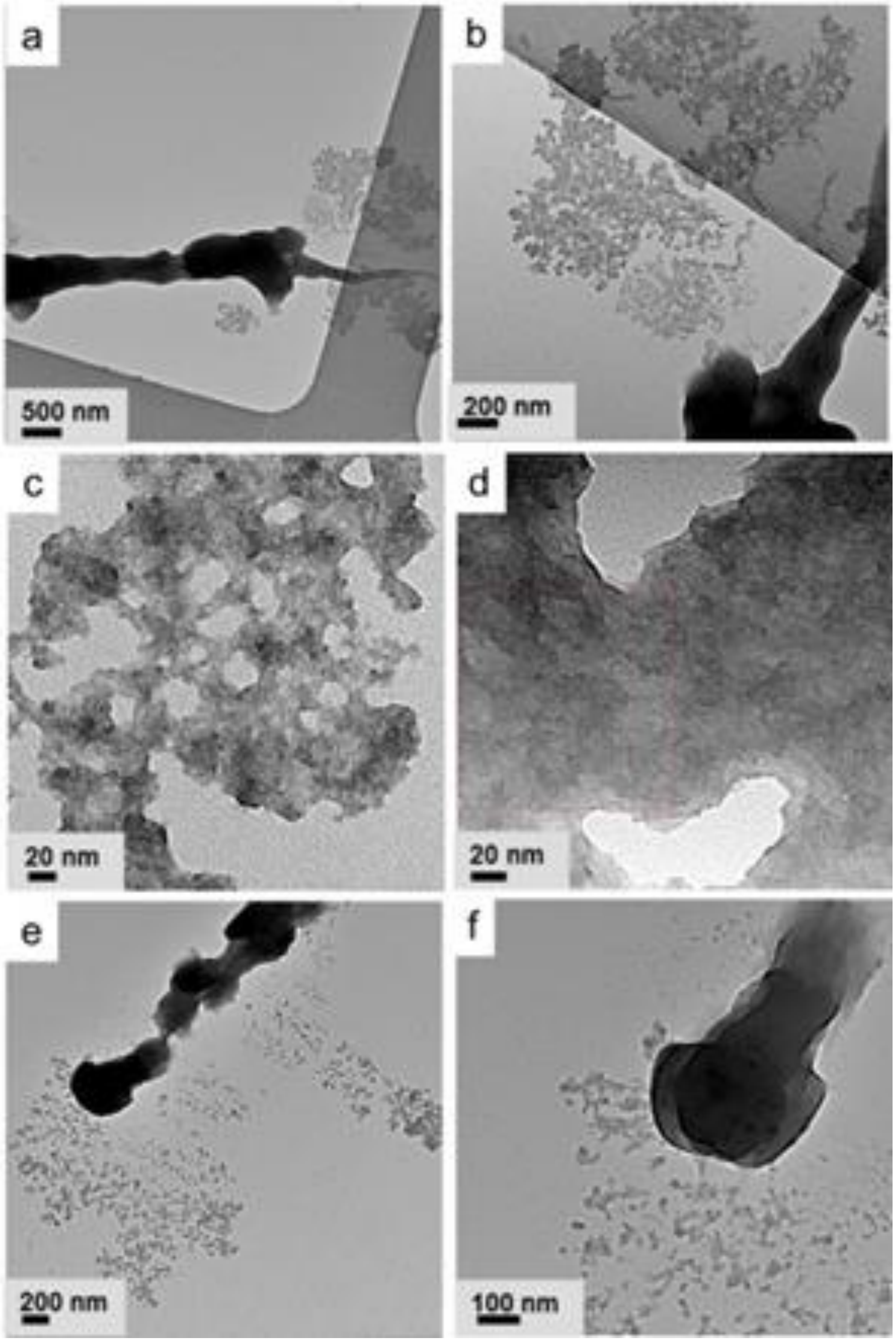

Figure S3.2. 12 hours incubation. (a) The S-layer-free cell is situated at left with discarded mineralized bacterial membrane on the right. (b) Enlarged view of cell showing that the bacterial membrane is in direct neighborhood and thus contacts to the cell. (c, d) At higher magnification, the nanoporous structuring of the bacterial membrane becomes evident. (e, f) Cell with discarded but not fully mineralized bacterial membrane reveals that the membrane already dropped at the beginning of the mineralization process.

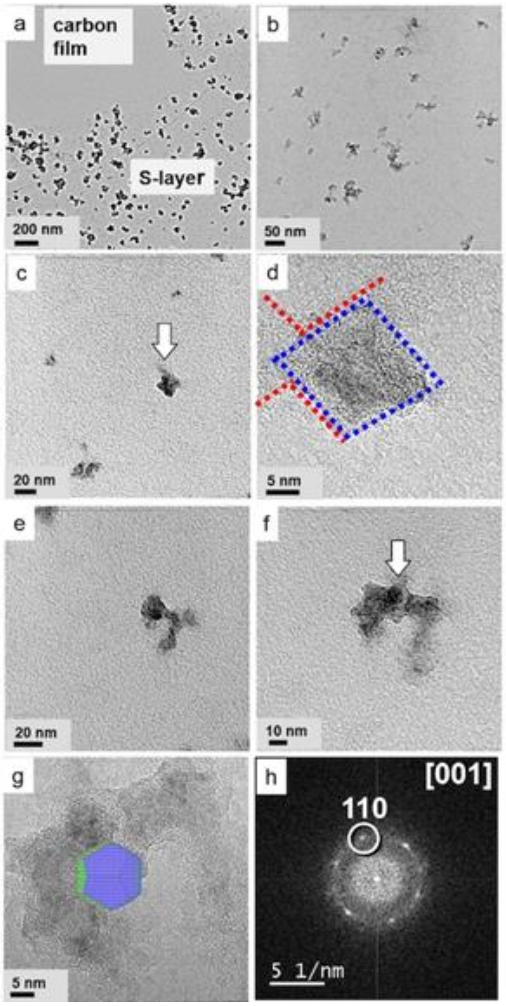

Figure S3.3. 12 hours **i**ncubation. Zoom series of TEM images of mineralized and reassembled S-layer of *G. stearothermophilus* incubation *in vitro* at 4° C. The $CaCO_3$ crystals appear as dark, thus electron dense aggregates. (a) At the edge of the S-layer (bottom) only the S-layer is mineralized whereas the carbon support remains untouched (top left). (b) Zoomed image shows different early stages of mineralization. (c) The rhombic aggregate may indicate an initial step of mineralization where an intersection (corner) of near-ordered structure elements (bars) with the size of the S-layer unit cell is mineralized. The particle has two bars starting to grow from the upper (white arrow) and the right corner. (d) High-resolution of the aggregate situated in the center of (c). The calcium carbonate aggregate is about 20 nm x 25 nm in size and consists of nuclei of calcite crystals with about 3-5 nm. (e) Another rhombic motif is discernable with a size of about 40 nm. (f) Image of larger "half"-rhombus with size 50 nm size. (g) High-resolution of (f) reveals massive mineralization at the left corner. (h) Corresponding FFT from central part indicates calcite in [001] orientation.

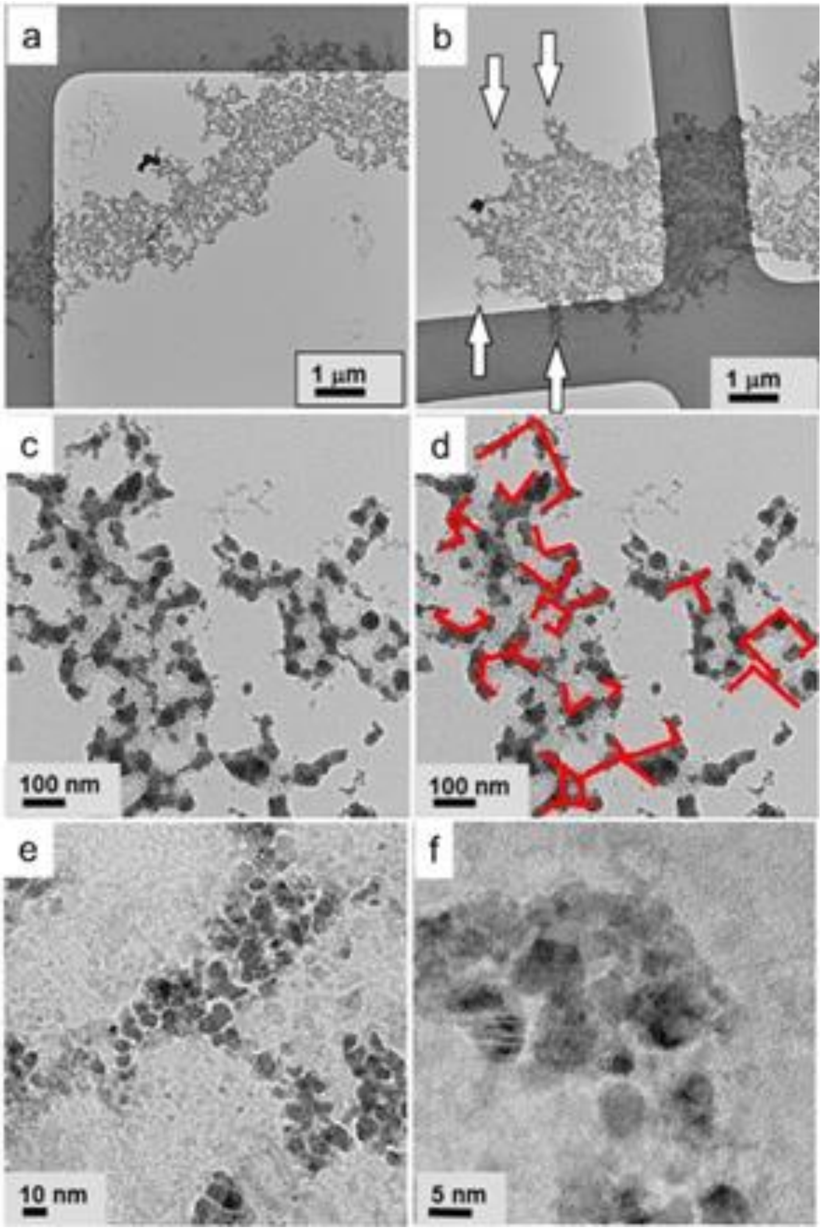

Figure S3.4. TEM images of mineralized bacterial membrane of *G. stearothermophilus*: discarded and outstretched membrane after 24 hours incubation at 4 °C. (a) Completely mineralized and discarded membrane about 9 microns in length and 1.5 microns in width reflecting the shape of the cell. (b) Zoomed image of one end of a mineralized membrane displaying nanoporous pattern and thin bars (white arrows) serving as interconnecting elements for the enwrapped cell. (c) Further enlargement reveals complex nested mineralization patterns. (d) Overlay of image (c) with geometric forms, which resemble of parts of nested rhombi with different sizes and orientations. (e, f) High-resolution images show nanosized calcite and other calcium carbonate crystals as constituents of the mineralized structures.

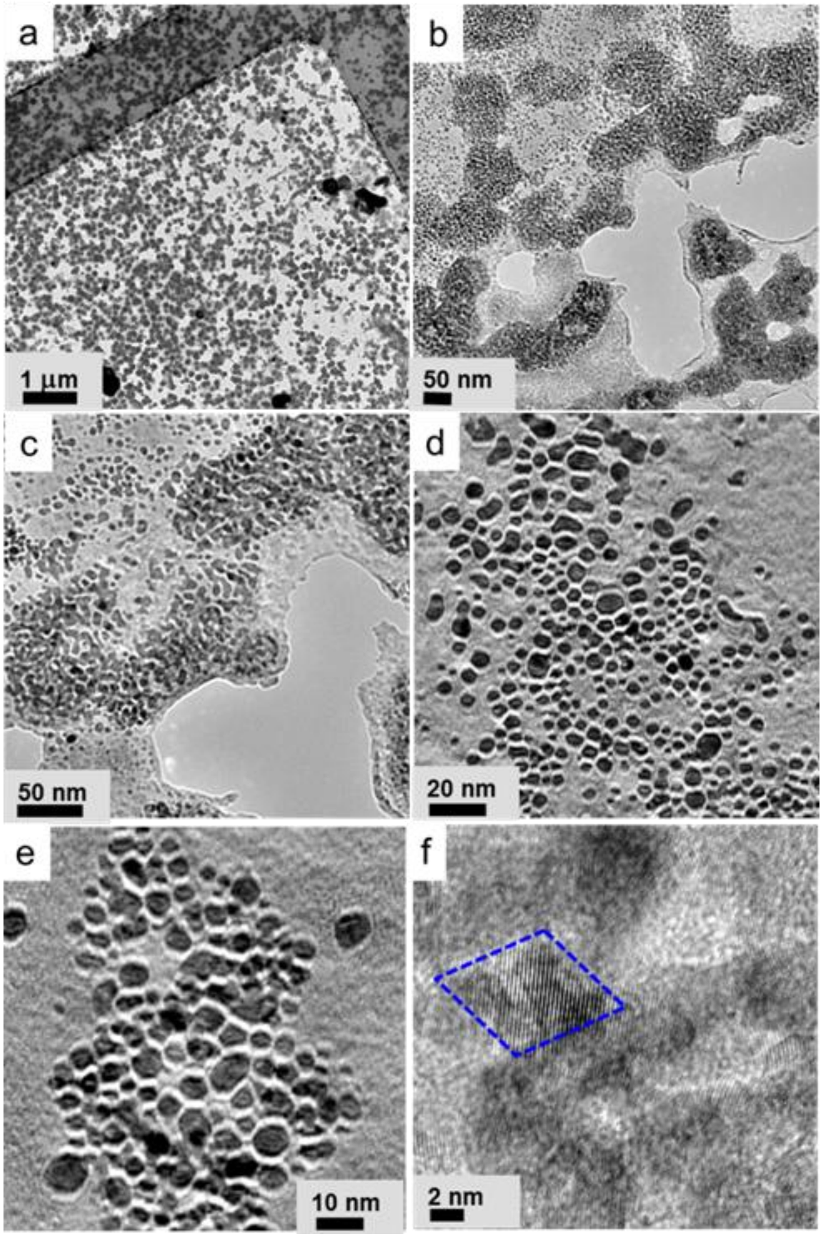

Figure S3.5. 24 hours incubation. (a, b) The reassembled S-layer is highly mineralized indicated by electron dense and thus dark agglomerates with several 100 nm in diameter. (c) Within the aggregates, a fine network is visible indicating compartments. (d, e) In the less mineralized part of the S-layer the individual compartments are clearly imaged. They are separated from each other by ultrathin protein walls with a thickness of 0.5 - 1 nm. Compartment diameters vary from 3 -12 nm. The patterns remind on Voronoi tiling. (f) High-resolution image showing a rhombic calcite crystal marked by the blue frame area.

Supplement S4: Fourier analysis of the mineralized calcium carbonate polymorphs of a reassembled S-layer of *G. stearothermophilus* after 12 hours.

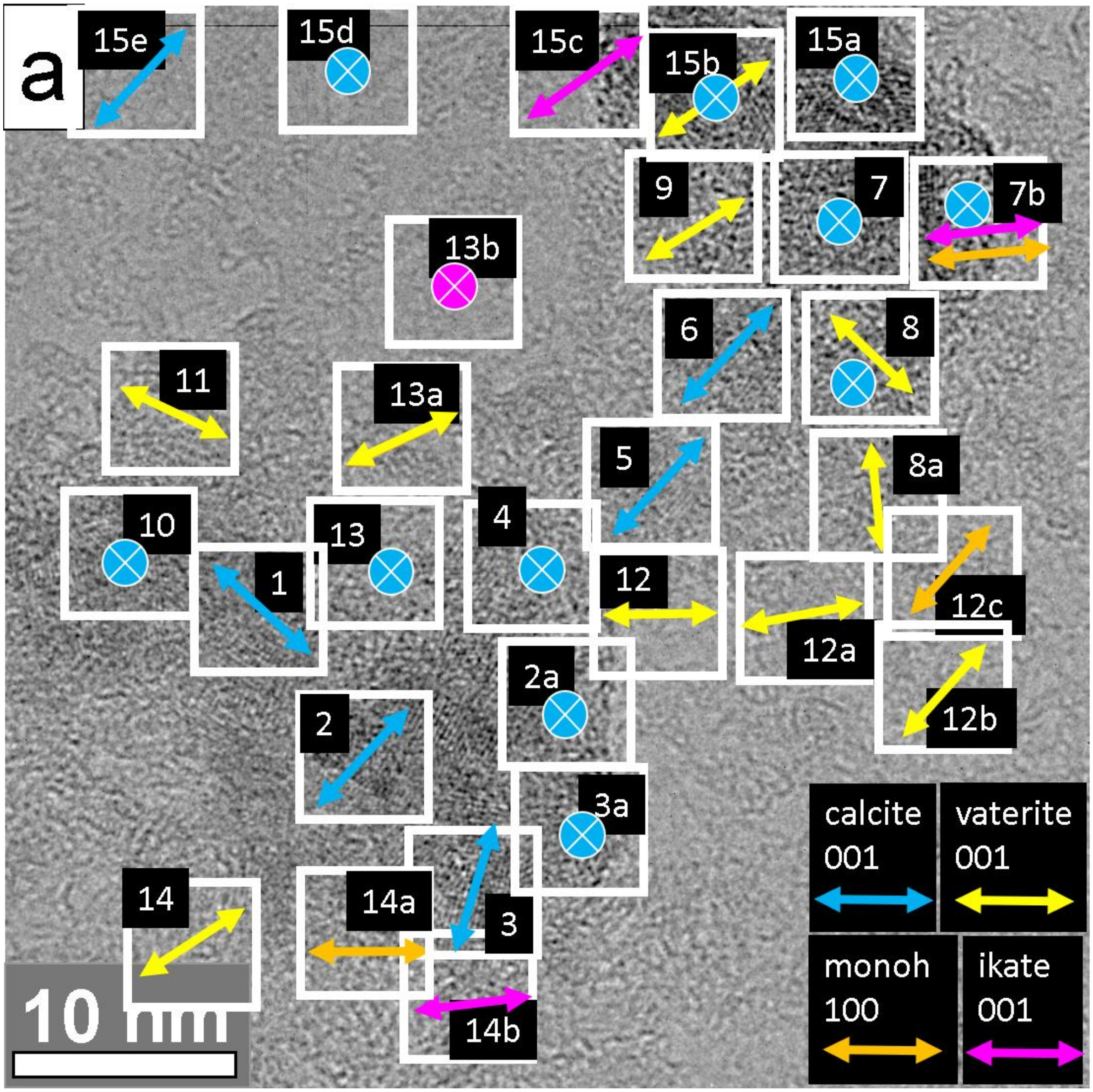

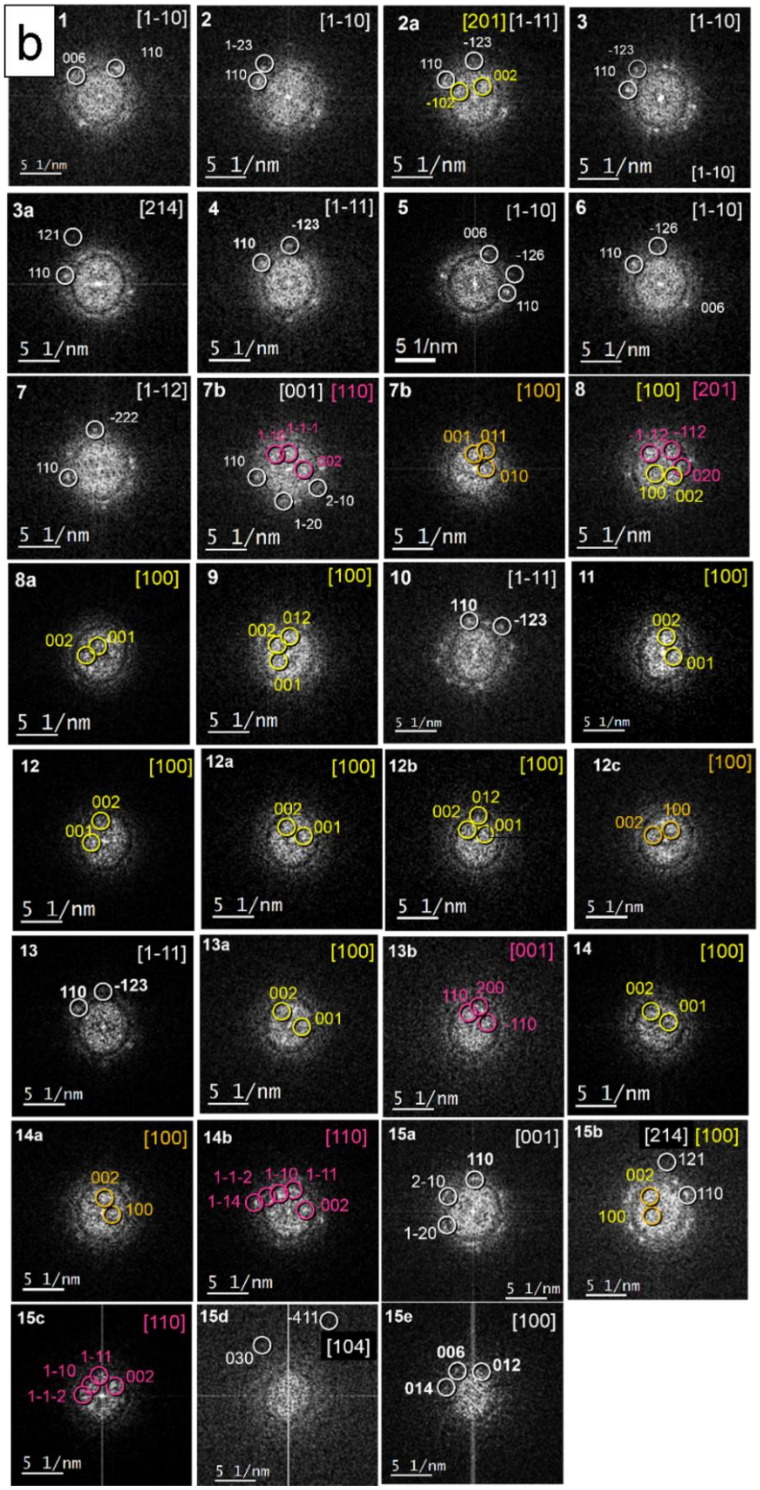

Figure S4.1: (a) High-resolution-TEM image of nanocrystals on the reassembled S-layer. (b) Fourier analysis of the nanocrystals indicates different orientations in (a) of calcite with blue arrows corresponding to *c*-axis of calcite. The c-axis of vaterite is indicated in yellow. The *a*-axis of monohydrocalcite is marked in orange and the *c*-axis of ikaite in pink.

The orientations of the faces in the dominating growth directions of the various polymorphs summarized in Table S4.1 show that the main part of crystals is grown in a kinetic regime (K). Only in the spots 15d with calcite (104), 3a and 15b with calcite (214), 7b and 15a with calcite (001), as well as 13b with ikaite (001), the morphology has already approached a constrained thermodynamic equilibrium geometry (T). The faces with calcite (104), and with ikaite (001) are faces of minimum surface energies. The normal of the face with calcite [001] deviates by 4.18°, and the normal of the face with calcite [214] by 8.25° from normal of the face with calcite [104].

1	(1 -1 0) calcite K	11	(1 0 0) vaterite K
2	(1 -1 0) calcite K	12	(1 0 0) vaterite K
2a	(1 -1 1) calcite K<br>(2 0 1) vaterite	12a	(1 0 0) vaterite K
3	(1 -1 0) calcite K	12b	(1 0 0) vaterite K
3a	(2 1 4) calcite T	12c	(1 0 0) mhc K
4	(1 -1 1) calcite K	13	(1 -1 1) calcite K
5	(1 -1 0) calcite K	13a	(1 0 0) vaterite K
6	(1 -1 0) calcite K	13b	(0 0 1) ikaite T
7	(1 -1 2) calcite K	14	(1 0 0) vaterite K
7b	(0 0 1) calcite T	14a	(1 0 0) mhc K
7b	(1 0 0) mhc K	14b	(1 1 0) ikaite K
7b	(1 1 0) ikaite K	15a	(0 0 1) calcite T
8	(1 0 0) vaterite K<br>(2 0 1) ikaite K	15b	(2 1 4) calcite T
8a	(1 0 0) vaterite K	15c	(1 1 0) ikaite K
9	(1 0 0) vaterite K	15d	(1 0 4) calcite T
10	(1 -1 1) calcite K	15c	(1 0 0) calcite K

Table S4.1. Dominating growth directions of the calcium carbonate crystals in the spots shown in Figure S4.1.

References

(1) Saeed, F., Uddin, F., Sultan, H. Thermodynamic study of monovalent and divalent cations in mixed solvent system by conductance method. *Phys. Chem. Liq.* **2007**, *45*, 313–321.

(2) Registration Dossier ECHA. EC number: 207-838-6.CAS number 497-19-8: Sodium carbonate dissociation constant. Hg. v. ECHA.

(3) Nakayama, F. S. Sodium Bicarbonate and Carbonate Ion Pairs and Their Relation to the Estimation of the First and Second Dissociation Constants of Carbonic Acid. *J. Physi. Chem.* **1970**, *74*, 2726–2728.
